# Space-use patterns highlight behavioural differences linked to lameness, parity, and days in milk in barn-housed dairy cows

**DOI:** 10.1371/journal.pone.0208424

**Published:** 2018-12-19

**Authors:** Jorge A. Vázquez Diosdado, Zoe E. Barker, Holly R. Hodges, Jonathan R. Amory, Darren P. Croft, Nick J. Bell, Edward A. Codling

**Affiliations:** 1 Department of Mathematical Sciences, University of Essex, Colchester, Essex, United Kingdom; 2 Writtle University College, Chelmsford, Essex, United Kingdom; 3 Centre for Research in Animal Behaviour, College of Life and Environmental Sciences, University of Exeter, Exeter, Devon, United Kingdom; 4 Royal Veterinary College, Hatfield, Hertfordshire, United Kingdom; University of Illinois, UNITED STATES

## Abstract

Lameness is a key health and welfare issue affecting commercial herds of dairy cattle, with potentially significant economic impacts due to the expense of treatment and lost milk production. Existing lameness detection methods can be time-intensive, and under-detection remains a significant problem leading to delayed or missed treatment. Hence, there is a need for automated monitoring systems that can quickly and accurately detect lameness in individual cows within commercial dairy herds. Recent advances in sensor tracking technology have made it possible to observe the movement, behaviour and space-use of a range of animal species over extended time-scales. However, little is known about how observed movement behaviour and space-use patterns in individual dairy cattle relate to lameness, or to other possible confounding factors such as parity or number of days in milk. In this cross-sectional study, ten lame and ten non-lame barn-housed dairy cows were classified through mobility scoring and subsequently tracked using a wireless local positioning system. Nearly 900,000 spatial locations were recorded in total, allowing a range of movement and space-use measures to be determined for each individual cow. Using linear models, we highlight where lameness, parity, and the number of days in milk have a significant effect on the observed space-use patterns. Non-lame cows spent more time, and had higher site fidelity (on a day-to-day basis they were more likely to revisit areas they had visited previously), in the feeding area. Non-lame cows also had a larger full range size within the barn. In contrast, lame cows spent more time, and had a higher site-fidelity, in the cubicle (resting) areas of the barn than non-lame cows. Higher parity cows were found to spend more time in the right-hand-side area of the barn, closer to the passageway to the milking parlour. The number of days in milk was found to positively affect the core range size, but with a negative interaction effect with lameness. Using a simple predictive model, we demonstrate how it is possible to accurately determine the lameness status of all individual cows within the study using only two observed space-use measures, the proportion of time spent in the feeding area and the full range size. Our findings suggest that differences in individual movement and space-use behaviour could be used as indicators of health status for automated monitoring within a Precision Livestock Farming approach, potentially leading to faster diagnosis and treatment, and improved animal welfare for dairy cattle and other managed animal species.

## Introduction

Globally, lameness is one of the key health and welfare issues that affects intensive dairy farms, particularly for herds that are housed indoors permanently or semi-permanently [1–3]. In the UK alone, the estimated cost to the dairy industry of treatment, lost milk yield, and lost fertility is over £128 million per annum [4]. Prompt treatment of lameness can reduce severity and the number of required treatments [5], hence reducing financial costs and the duration and impact of pain for the individual animal. However, early detection of lameness remains a problem as many farmers may underestimate the prevalence of lameness within their herd [2], identify and treat cows later than might be optimal [5], or time constraints may mean they are unable to undertake time- and labour-intensive mobility monitoring [6]. Increasing intensification of farming practices means that these detection issues are likely to become even more problematic in larger dairy herds. Hence, there is a need for systems which can automatically detect lameness at an early stage without the need for time-consuming mobility observations of individual animals. Recent attempts to use automated systems to detect lameness have relied upon the identification of abnormal gait using load cells, pressure sensitive mats, computer vision or accelerometers [7,8], but the uptake of such technology on farms has been limited due to both costs and practical effectiveness in the working farm environment. More generally, within Precision Livestock Farming approaches [9], a range of behavioural measures have been suggested as potential indicators of health status and disease for monitoring managed animal species. Lameness is known to cause pain and walking difficulty in affected cows [10,11], and this may influence how they move and use the available space within the barn. However, existing studies that have linked the lameness status of individual dairy cows to their space-use behaviour have been restricted to small spatial scales (i.e. at the level of individual stalls) [12].

In the wider movement ecology context, animal movement and space-use behaviour is known to be influenced by landscape characteristics such as the location of water or food resources, habitat type and vegetation cover [13], as well as local topographic features such as the gradient of a hillside [14]. For example, grazing animals are known to move to locations with higher quantities of grass resources or where there is better quality of nutrients [15]. By adapting to their environment, animals can hence visit favourable areas more than others [16,17]. In a limited home range, an animal may repeatedly visit certain locations [18] or actively spend more time in specific areas leading to different levels of space-use intensity. For an individual animal, the level of similarity in its space use at different time points can be calculated and the level of site fidelity quantified [19–22]. In the specific context of pasture-based cattle, [23] showed how spatial overlap between domesticated cattle and wild buffalo was linked to the gradient of available resources, [24] demonstrated how concentrate supplement can modify the feeding behaviour of grazing cows in high mountain pastures, while [25] considered how spatial interactions between cattle and wild boar could potentially facilitate cross-species disease transmission.

Understanding how illness or welfare status may affect animal movement behaviour, space-use, and interactions with the local landscape could potentially provide extremely useful insights and indicators for monitoring and managing a range of animal species [9]. Lameness, mastitis and ketosis (metabolic disorder) are all important diseases of dairy cattle that have been shown to affect feeding and lying behaviour [26–32], and in this context, the use of cow-mounted accelerometers to measure cow behaviour is well established [8,33–35]. However, despite these extensive studies highlighting the links between disease and dairy cow behaviour, an automated method for disease detection based on behavioural observations is still lacking. A major issue with any automated approach is the complex interplay between health status and other potential confounding factors such as age, parity, or stage of lactation [36,37], that lead to individual behavioural differences. A recent study investigated a range of possible behavioural indicators of health status in dairy cattle including lying, locomotion, feeding and rumination activities, in addition to brush and concentrate feeder visits and milking order [29]. Although there were differences between lame and non-lame cows, [29] also reported a high variability across individual animals and predictor variables overlapped between these groups.

The use of spatial tracking systems for monitoring dairy cattle is less well developed than accelerometer-based systems, but they have the potential to provide additional important behavioural information about movement and space-use in individual animals. With pasture-based animals, tracking is possible with standard Global Positioning Systems (GPS) [38,39], but for indoor barn-housed dairy cows alternative systems are needed. Real-Time Location Systems (RTLS) are a recent new development in the application of radio frequency technology with great potential for use in livestock agriculture. They have been tested and validated for indoor spatial tracking of dairy cows [28,40–42], and have subsequently been used to predict time budgets of behavioural activities [43], to determine the probability of cattle undertaking feeding or drinking [44], or to detect behavioural changes related to oestrus [45]. However, to date there have been no studies that have reported how differences in space-use behaviour within a commercial barn may be directly linked to the lameness status of individual dairy cows.

In this paper we present results from of an analysis of a tracking data set containing nearly 900,000 recorded spatial locations obtained over five days from a cross-sectional study group of ten lame and ten non-lame barn-housed dairy cows using a wireless positioning system. We determine measures of space-use behaviour within the barn for each cow, and using linear models we demonstrate where lameness status, parity and days in milk have a significant effect on the observed space-use measures. We demonstrate how observable differences in space-use patterns can be used directly within a simple predictive model to accurately determine the lameness status of individual cows. The methodology and approach described within the study could be adapted to study space-use behaviour in other commercially managed or wild animal species.

## Materials and methods

The study was carried out in strict accordance with the UK Animal Welfare Act (2006). The study was reviewed and approved by the Royal Veterinary College Ethics and Welfare Committee under the unique reference number 2012 1223. The study was non-invasive and the collars used were similar to those in standard industry use. Lame cows were managed according to the farm’s animal health plan and all animals were monitored daily whilst in the study in order to identify any potential welfare issues which needed addressing.

### Data collection

**Fig 1:**
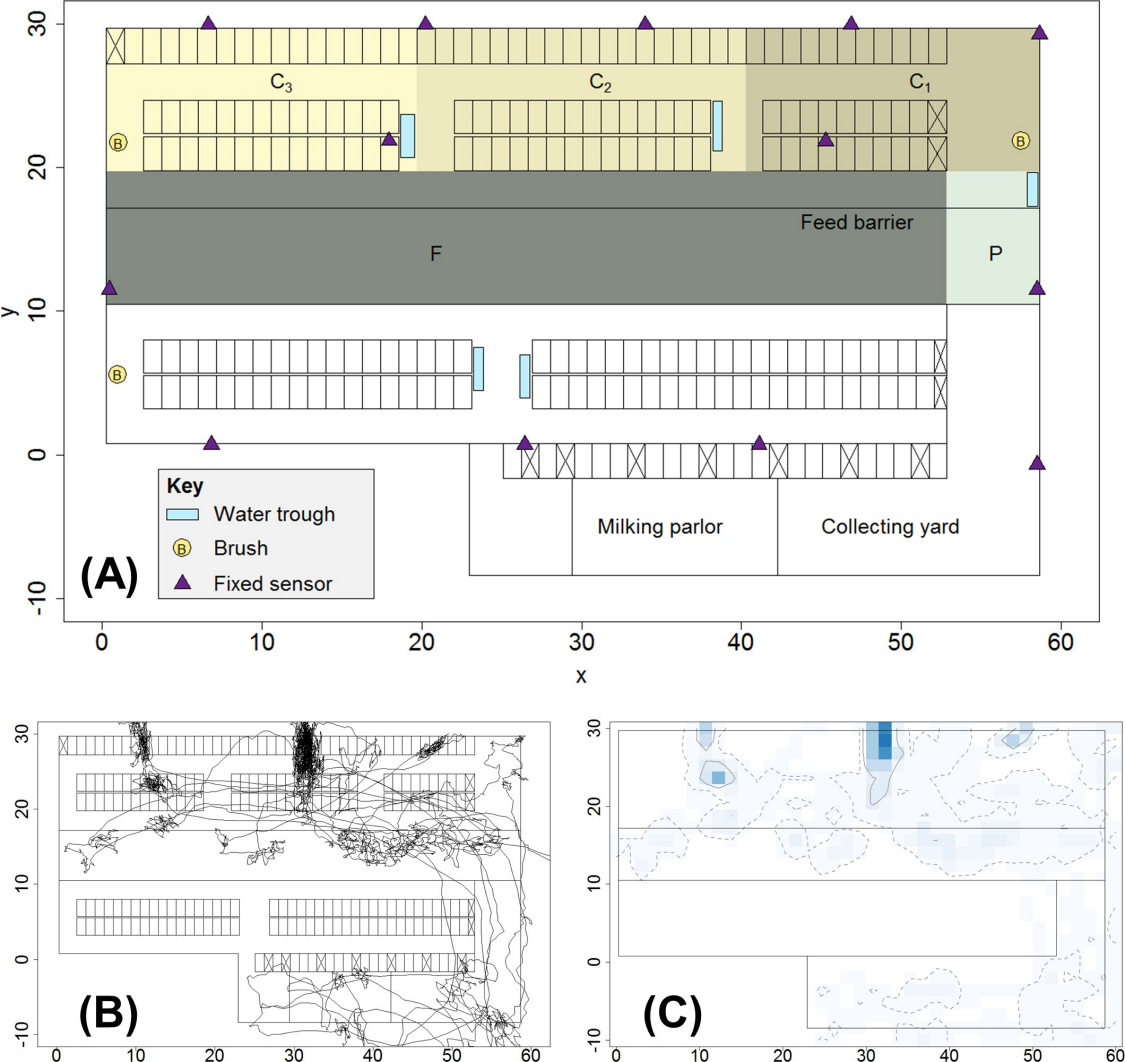
Schematic map of barn and examples of cow movement trajectory and space-use intensity. (A) Schematic map of barn indicating features and areas of interest. Fixed sensors were positioned on the barn walls to aid tracking of mobile cow-mounted sensors. Areas C_1_, C_2_ and C_3_ are zones defined to correspond to the three main cubicle blocks in the upper barn area (C_T_ is the total cubicle area corresponding to the union of C_1_, C_2_ and C_3_); area F corresponds to the feeding zone and includes space either side of the feed barrier; area P is a passageway allowing access from the upper barn area to the collecting yard and milking parlour. (B) Example of a cow trajectory (cow 1078, day 5) produced by smoothing the raw sensor-collected data using a simple moving average over a 15 time-step (2 minute) moving window. (C) Example space-use intensity plot (cow 1078, day 5) produced by overlaying a 1.5m^2^ square grid onto the map of the barn and counting the cells in which trajectory points are found. Darker colours correspond to higher space-use intensity. The 95% and 50% isopleths are respectively indicated by the dashed and solid contour lines. Note that the plot shows space-use data from the full barn for illustrative purposes; results in the main paper are for location data from the upper barn only, see Fig 2.

The study was undertaken over 5 successive days in January 2014 on a commercial dairy farm in Essex, UK. A total of 210 Holstein pedigree cows were housed in a rectangular free-stall barn measuring 30m by 60m. The cows were split into high yield (120 cows) and low yield (90 cows) groups, separated by a central feed alley (Fig 1A). The high yield group were housed in the upper part of the barn and had access to 120 free-stalls, and linear feed space of 0.43m per cow. The milking parlour and collecting yard were situated in the lower part of the barn, with a connecting return passage positioned on the right-hand side of the barn (Fig 1A). All cows were fed a commercial total mixed ration (TMR) and milking took place three times a day (05:00hrs, 13:00hrs, 21:00hrs).

To explore the effect of lameness, and possible confounding factors such as parity and days in milk, on cow space-use and behaviour, a cross-sectional study design was used. Prior to the study, all cows in the high yield group were locomotion scored at the exit to the milking parlour using the 4-point AHDB Dairy Mobility Score [46] by ZB (where 0 = sound and 3 = severely lame), and re-scored in the main barn by HH the following day. Cows which were known to have had a health incident in the previous three months, including foot lesions and mastitis treatments, were excluded. Two study groups (10 lame cows and 10 non-lame cows) were selected based on their mobility scores, and to match yield and parity where possible (S1 Table). It should be noted that only cows with a mobility score of 2 (‘lame’) were included in the lame group; score 3 cows (‘severely lame’) were not included due to a low number of cows with this score within the herd, and for ethical reasons (the selected cows would not be treated until the end of the study). Selection of the cows was made without any prior knowledge of their space-use behaviour. Individual parity ranged from 1 to 6 years (mean = 3.25, s.d = 1.44), and for the current parturition period, days in milk (DIM) ranged from 44 to 220 (mean = 125, s.d. = 51.3, and mean daily milk yield (in litres) ranged from 28.7 to 58.4 (mean = 42.5, s.d. = 6.88), see S1 Table. At the end of the study period all cows were clinically inspected for lameness and foot trimming was carried out where appropriate.

The selected cows were fitted with wireless sensors (Ominsense Series 500 Cluster Geolocation System [28,35,47,48]; www.omnisense.co.uk/), to track spatial location in the upper area of the barn. The Series 500 sensors form a RTLS wireless network able to compute relative spatial locations in (*x*, *y*, *z*) coordinates of each individual sensor within the system using the arrival time of periodic messages sent from each node to its neighbours to triangulate distances (note that in this study, cows were restricted to a single elevation, so only the (*x*, *y*) coordinates were used). Thirteen sensors were attached to known fixed positions around the barn and a further eight were positioned within the adjacent collecting yard and milking parlour to improve network coverage and triangulation measurements (Fig 1A). Validation of sensor precision and accuracy within this specific barn environment has been reported previously in [28]. The sensors were found to perform well for spatial tracking of individual cows, although performance was slightly worse than the commercially advertised specification (95% of measurements within 2 m of ground truth; Omnisense Ltd.), which is likely due to metal features within the barn environment disrupting the sensor signals [28]. The sensors were mounted on cows using a neck collar that incorporates a counterweight to keep the sensor in a stable position at the top of the neck [28,35].

Location data were collected continuously for 24 hours per day over the 5 days of the study using a 0.125Hz sample rate, leading to a theoretical maximum of 54,000 location data points being collected per cow over the duration of the study. However, location data during the three daily milking events, each lasting approximately 90 minutes when the cows left the upper barn area, were excluded as cow movement and space-use behaviour was constrained by human interventions at these times. In addition, some further minor data loss occurred when sensors occasionally suffered battery failure before being replaced, or when sensor error seemingly placed a cow outside the barn (any such coordinates were removed from the analysis). In total, 876,621 location data points (81% of the theoretical maximum) were collected in the upper barn area and used in the following data analysis. The mean number of location data points collected per day across all cows was 8767 (median = 8930), and the minimum average number of data points collected for a single cow over the 5 days of the study was 8175 data points per day.

The sensor recorded raw location data were smoothed to remove outliers using a simple moving average (SMA) over a two-minute moving window (i.e. 15 data points at the 0.125Hz sampling rate; Fig 1B). Basic movement and space-use measures calculated directly from the smoothed sensor location data include the total distance moved per hour, and the mean *x* and mean *y* locations.

A basic analysis of this data set was described in [28], where spatial location data were used alongside accelerometer data in a decision tree algorithm to classify cow behaviour as either ‘feeding’, ‘non-feeding’, or ‘out of the pen for milking’. Differences in the daily activity budgets between lame and non-lame cows were highlighted, with lame cows spending significantly less time feeding. However, [28] only considered daily behavioural time budgets and did not directly consider differences in space-use measures or site fidelity between the lame and non-lame groups as we do in more detail here.

### Space-use intensity and the utility distribution

Animal space use intensity can be quantified from location data using a utility distribution (UD) for each individual animal [49–51]. In many movement data sets, spatial locations are only recorded at low temporal sampling resolutions or there may be missing data, and a range of methods have been developed to estimate the UD in such cases [52]. These include kernel density estimation [50,53], the Brownian bridge movement model (BBMM) [15,54,55], step-selection analysis [56], and state space models [57]. However, since our location data are collected at high temporal sampling resolution (0.125Hz) with very few missing data points, and we calculate the UD on a daily basis over a confined spatial area, a simple cell-counting method is much more computationally efficient and will give similar results [52,58].

To determine the daily UD for each individual cow in our data set we overlay a virtual 40 x 13 square grid of 1.5m x 1.5m (= 2.25m^2^) cells onto the upper barn area (0 ≤ *x* ≤ 60, and 10 ≤ *y* ≤ 30 in Fig 1A). The cell size is chosen to be slightly larger than the known sensor precision and to roughly correspond to the area that can be occupied by a single cow. For each cow, each of the smoothed (*x*, *y*) coordinate locations in the upper barn area are assigned to the relevant grid cell and the count for that cell is increased by one. Any coordinate locations lying outside the upper barn area are removed (corresponding to milking periods or when sensor error resulted in a location outside the barn). The final daily UD is then rescaled to form a discrete probability distribution that sums to 1, by dividing all individual cell counts by the total cell count across all cells (Fig 1C).

To explore relative space use intensity we use the UD to determine the mean daily proportion of time spent in the upper barn area in specific biologically relevant areas of interest (see Fig 1A): feeding area (F: 0 ≤ *x* ≤ 53, 10 ≤ *y* ≤ 20); full cubicle area (C_T_: 0 ≤ *x* ≤ 60, 20 ≤ *y* ≤ 30); right-hand cubicle area (C_1_: 40 ≤ *x* ≤ 60, 20 ≤ *y* ≤ 30); central cubicle area (C_2_: 20 ≤ *x* ≤ 40, 20 ≤ *y* ≤ 30); and left-hand cubicle area (C_3_: 0 ≤ *x* ≤ 20, 20 ≤ *y* ≤ 30). The feeding area, F, is defined on either side of the physical feed barrier marked in Fig 1A, and does not span the entire width of the barn. A small area on the right-hand side of the barn, marked as P on Fig 1A (P: 53 ≤ *x* ≤ 60, 10 ≤ *y* ≤ 20), serves as a passage and return to the milking parlour. The cubicle areas include fixed cubicle blocks, where cows are able to lie down in individual cubicles (stalls), as well as interconnecting passageways (Fig 1A).

**Fig 2:**
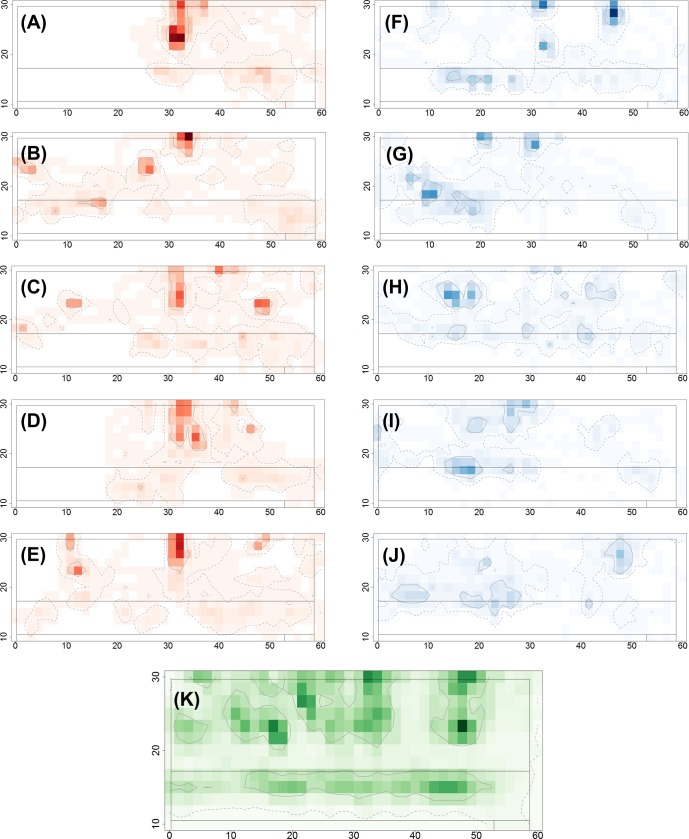
Space use intensity plots illustrating typical utility distributions over the five days of the trial. Plots are shown for (A-E) a single lame cow (cow 1078), and (F-J) a single non-lame cow (cow 2179), for each of the five days of the study. The space-use intensity UD is calculated by overlaying a 1.5m x 1.5m square grid (40 x 13 cells) onto the upper barn area only and counting the cells in which the smoothed trajectory points for each cow occur for each day of the trial. Darker colours correspond to higher space-use intensity. The 95% and 50% isopleths (corresponding to the full and core ranges for movement within the upper barn area only) are respectively indicated by the dashed and solid contour lines. (K) Space use intensity plot calculated in the same manner as above but using the aggregated data from all 20 cows over all 5 days of the study.

In animal home range analysis, the 50% isopleth (the contour line which can be drawn on the UD corresponding to the highest density cells that cumulatively account for 50% of the total observed density) is often considered as the ‘core’ home range of the animal as it contains those cells where the animal spends the most amount of time [59–63]. Similarly, the 95% isopleth is considered to be the ‘full’ or largest extent of the home range; cells lying outside the 95% isopleth are usually assumed to correspond to noise in the data or to areas only very infrequently visited [59–63]. For our location data, we truncate and rescale the UD at both the 95% isopleth (full range) and 50% isopleth (core range) levels (Fig 1C; Fig 2). We determine the mean daily size of the full and core ranges for each cow, measured in terms of the number of virtual cells lying inside the relevant isopleth level.

### Site fidelity

By comparing the level of overlap or similarity of UDs at different time-points it is possible to determine how the level of consistency of space-use, or site fidelity, of an animal may change over time [19–21]. Assuming two different discrete UDs that have both been rescaled as probability distributions, the Bhatacharyya coefficient (or Bhattacharyya's affinity) is a simple way to compare the level of similarity or overlap of the UDs [59,64,65]:

$$O_{t_{1}t_{2}}=\sum_{q\in Q}\sqrt{U_{t_{1}}(q).U_{t_{2}}(q)},$$ (1)

where *q* represents each discrete cell in the spatial grid and $U_{t_{i}}(q)$ is the probability mass for that cell at time *t*_*i*_. The Bhattacharyya coefficient ranges from 0 (no overlap) to 1 (full overlap). We calculate the Bhattacharyya coefficient for each cow using the UD across the full upper barn areas compared across successive days within the study. The space-use similarity score of smaller UDs corresponding to site fidelity in the feeding area (F) only, and the cubicle area (C_T_) only, are also calculated. We determine an overall measure of the similarity score of corresponding UDs over the 5 days of the study (i.e. an overall measure of site fidelity) for each cow by determining the average Bhattacharyya coefficient calculated from each pair of consecutive days:

$$\bar{O}=\frac{1}{4}\sum_{i=1}^{4}O_{t_{i}t_{i+1}}$$ (2)

To check the robustness of results to the averaging procedure used, we also considered two further approaches: averaging the Bhattacharyya coefficient over all possible combinations of pairs of days within the study (10 possible unique pairs in total), and a similar approach but where each pair of days is weighted according to the metric distance between the days before taking the average. However, site-fidelity results were very similar using all three approaches and hence we only report results from the simplest method here.

### Statistical analysis of space-use

Although our main interest in this study is to explore behavioural differences between lame and non-lame cows, it is important to also consider potential confounding factors. Hence in the statistical analysis we consider three predictor variables: ‘lameness’ (*L*, assigned to a binary variable with 1 as lame and 0 as non-lame), ‘parity’ (*P*), and ‘days in milk’ (*D*, calculated over the current parturition only), see S1 Table. We also considered mean daily yield as a predictor variable but preliminary investigations showed that this had no effect and hence was not included in the subsequent analysis. As data were collected continuously over the 5 days of the study for all cows, with no specific management interventions on any days, we do not include ‘day’ as a predictor variable in our analysis (S2 File highlights no clear trends or differences by day between the lame and non-lame groups for any of the basic space-use measures considered). Similarly, as the study only lasts for 5 days, we do not have a long enough time period of data to consider changes in lameness status (or parity or DIM) during the study (although this may be possible in much longer studies).

In total, sixteen different space-use and site-fidelity dependent variables, *S*_*1*_ to *S*_*16*_, were considered: *S*_*1*_: mean distance moved per hour; *S*_*2*_: mean *x* coordinate; *S*_*3*_: mean *y* coordinate; *S*_*4*_: proportion of time spent in the feeding area (F); *S*_*5*_: proportion of time spent in the full cubicle area (C_T_); *S*_*6*_ *–S*_*8*_: proportion of time spent in each of the specific cubicle areas (C_1_, C_2,_ C_3_ considered separately); *S*_*9*_: mean size (in virtual cells) of the daily ‘full’ range (corresponding to the 95% isopleth of the UD); *S*_*10*_: mean size (in virtual cells) of the daily ‘core’ range (corresponding to the 50% isopleth of the UD); *S*_*11*_ *–S*_*16*_: site fidelity determined for each of three areas (full upper barn area, feeding area (F) only, and cubicle area (C_T_) only) for two different isopleth levels (full range = 95%; core range = 50%).

Statistical analysis was undertaken using model selection based on a multivariate linear (regression) model with the three predictor variables (lameness, *L*; parity, *P*; days in milk, *D*). Linear models corresponding to all possible combinations of the predictor variables and their interaction terms were fitted to each of the individual space-use measures, *S*_*1*_ to *S*_*16*_ in turn:

$$S_{i}=a_{0}+a_{1}L+a_{2}P+a_{3}D+\mathrm{interaction}\;\mathrm{effects},$$ (3)

where *α*_*n*_ are regression coefficients to be determined (*α*_0_ is the intercept). For each linear model, the Akaike Information Criterion (AICc; corrected for small sample sizes) was used to select the best relative fitting model for that space-use measure [66] (the lowest AICc score corresponds to the best fitting model). For the best fitting linear model, the *F*-statistic and associated *p*-value are then used to determine whether the model is a significantly better fit (at the 5% level) to the data than an intercept-only model (which does not include any of the predictor variables). Subsequently, the individual *p*-values corresponding to each regression coefficient, *α*_*j*_, are used to determine the significance (at the 5% level) of each predictor variable (and any interaction effects) within the linear model.

For the multivariate linear regression model to be valid the following assumptions must hold [67]. Firstly, there must be a linear relationship between the predictor variables and the dependent variables (we assume this implicitly during the analysis, and also check by examining the data visually in the output plots). Secondly, there must be no multicollinearity between the predictor variables. To test this, Variance Inflation Factor (VIF) scores were calculated for each predictor variable (*L*_VIF_ = 1.465776, *D*_VIF_ = 1.0714, *P*_VIF_ = 1.3877), and since no scores were higher than the threshold score of VIF >10, we conclude that there is not a high level of multicollinearity between our predictor variables [68]. Thirdly, the model residuals must be normally distributed; and finally, there must be no heteroscedasticity within the data [67]. For each fitted linear model, we test the residuals for normality using the Shapiro-Wilks test (S-W; 5% significance level) and for heteroscedasticity using the non-constant variance test (NCV; 5% significance level). Regression and model fitting were undertaken using the ‘glm’ and ‘AICc’ functions in R [69].

### Predictive model for lameness

To explore the potential predictive capability of the observed dependent variables to correctly classify lameness in individual cows we also consider a generalised linear regression model with logit link function of the form:

$$log\left(\frac{p}{1-p}\right)=b_{0}+b_{1}S_{1}+\cdots +b_{n}S_{n},$$ (4)

where *β*_*n*_ are regression coefficients to be determined (*β*_0_ is the intercept), *S*_*i*_ are the corresponding values of the observed dependent variables in the previous analysis, and *p* represents the estimated probability from the model that a cow is classified as lame. To avoid over-fitting the predictive model, we restrict the model selection choice to those dependent variables, *S*_*i*_, where one or more of the predictor variables were found to be significant in the previous analysis. As above, we determine the best relative fitting model using model selection via the Akaike Information Criterion score, AICc (corrected for small sample sizes) [66]. Regression and model fitting were undertaken using the ‘glm’ and ‘AICc’ functions in R [69].

## Results

### Space-use intensity and other basic space-use measures

Fig 2 shows illustrative daily space-use intensity UDs in the upper barn area for a single lame cow (cow 1078; Fig 2A–2E) and a single non-lame cow (cow 2179; Fig 2F–2J) over the 5 days of the study (individual plots for all cows and all days of the study are shown in S1 File). Fig 2K illustrates the aggregated space-use intensity UD for all 20 cows over all 5 days of the study and highlights areas of higher space-use intensity (i.e. inside the 50% isopleth) corresponding to the cubicle and feeding areas, with lower space-use intensity in the corridors and passageways.

**Fig 3:**
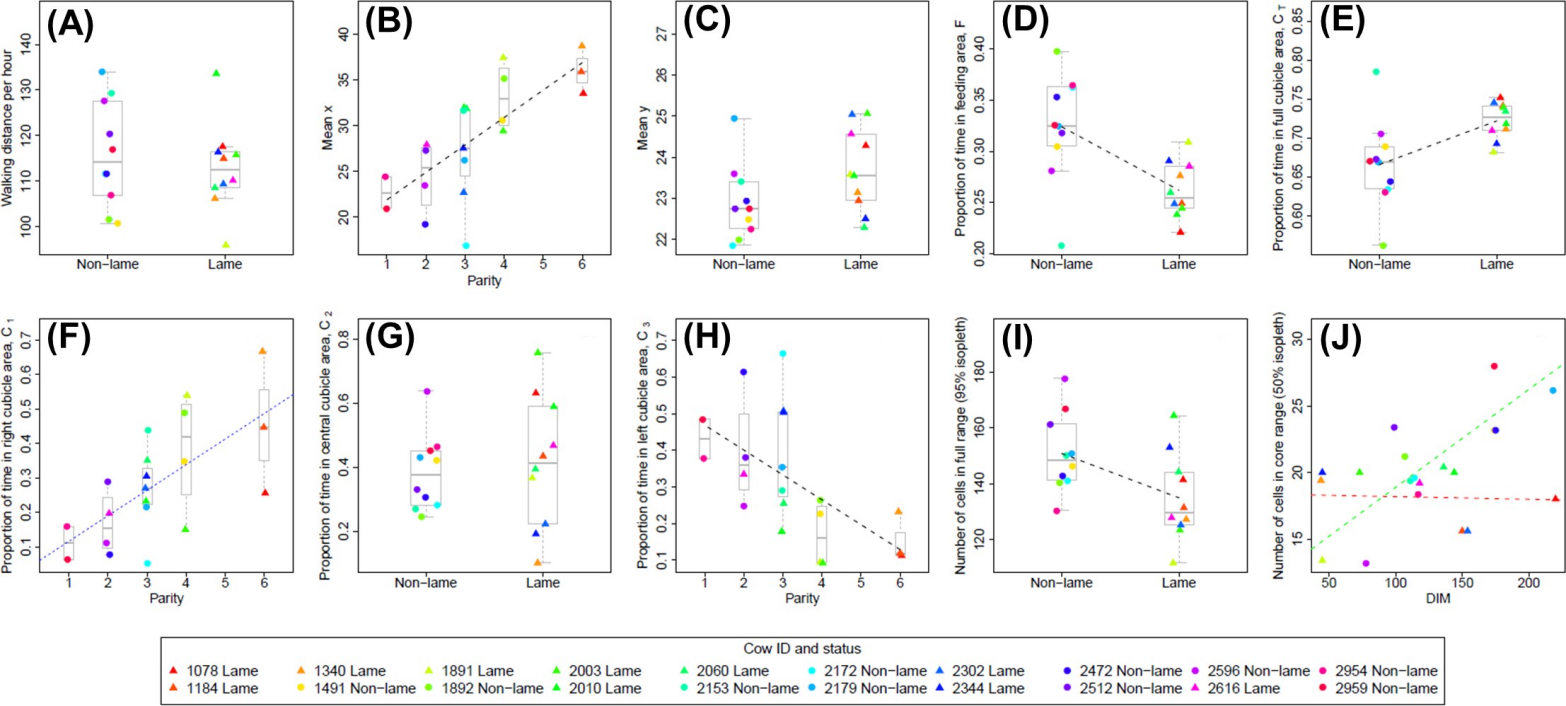
Plots showing relationship between significant predictor variables (lameness; parity; days in milk, DIM) and basic space-use measures. Data for each basic space-use measure, *S*_*1*_ to *S*_*10*_, are shown in plots (a) to (j) respectively, and are plotted against the most significant predictor variable determined from the model selection procedure (Table 1). Where none of the predictor variables are significant (at the 5% level) for a given model, the data is plotted for the lame and non-lame groups (a, c, g). Where appropriate, boxplots (with median line) are used to show the spread of the data for each level of the predictor variable (a–i). Individual data points are calculated as a mean average across all five days of the trial for each cow (S2 Table). Lame cows are plotted as filled triangles and non-lame cows as filled circles; the colours used to indicate each data point are fixed for each cow and are consistent across all plots (see legend). Where the best fitting linear model includes only a single predictor variable, the fitted regression line is shown as a dashed black line (b, d, e, h, i). In (f) the best fitting linear model includes both parity and DIM terms (Table 1); a regression line fitted only to the parity variable (the most significant predictor) is shown as a blue dashed line for illustrative purposes only. In (j), the best fitting linear model includes lameness, DIM, and an interaction term; regression lines fitted only to the DIM variable are shown for the lame group (red dashed line) and non-lame group (green dashed line) to illustrate the negative interaction of lameness with DIM.

Full results for each basic space-use measure (*S*_*1*_ to *S*_*10*_) at the level of each individual cow are given in S2 Table and are shown as individual data points in Fig 3. Model selection and subsequent analysis revealed that the predictor variables (lameness, parity, and days in milk) had statistically significant effects on a number of the space-use measures (Table 1). Lameness was found to have a significant negative effect on the proportion of time spent in the feeding area (*S*_*4*_, *p* = 0.004; Fig 3D), and conversely, had a significant positive effect on the proportion of time spent in the full cubicles area (*S*_*5*_, *p* = 0.011; Fig 3E). It should be noted however, that heteroscedasticity was found to be present in the residuals for this latter result (non-lame cows had significantly higher variance) and hence the result should be treated with caution. A weak positive effect of lameness on the mean *y* coordinate (*S*_*3*_, *p* = 0.08, Fig 3C) is consistent with these results given the relative location of the cubicle and feeding areas (Fig 1). Lameness was also found to have a significant negative effect on the full range size (95% isopleth), with non-lame cows having a larger number of cells in their full range (*S*_*9*_, *p* = 0.029; Fig 3I).

**Table 1:** Results of model selection for multivariate linear regression models using the predictor variables (lameness, parity and days in milk) for each of the space-use measures considered within the study.

Space-use measure	Best fitting linear model	AICc score	*F*-statistic (*p*-value)	Regression coefficient values (*p*-values)	Summary & notes
*S*_*1*_: mean distance moved per hour	*S*_1_ = *a*_0_ (Intercept only)	154.66	n/a	*a*_0_ = 114.49	No significance.
*S*_*2*_: mean *x* coordinate	*S*_2_ = *a*_0_ + *α*_2_*P*	120.42	***F* = 20.36** **(*p* < 0.001)**	*a*_0_ = 18.91 ***a***_**2**_ **= 2.997 (*p* < 0.001)**	*P* has a significant positive effect on *S*_*2*_.
*S*_*3*_: mean *y* coordinate	*S*_3_ = *a* + *α*_1_*L*	60.45	*F* = 3.44 (*p* = 0.08)	*a*_0_ = 22.90 *a*_1_ = 0.795 (*p* = 0.08)	(*L* has a weak positive effect on *S*_*3*_).
*S*_*4*_: proportion of time spent in the feeding area (F)	*S*_4_ = *a*_0_ + *a*_1_*L*	-64.59	***F* = 10.85** **(*p* = 0.004)**	*a*_0_ = 0.324 ***a***_**1**_ **= - 0.062 (*p* = 0.004)**	*L* has a significant negative effect on *S*_*4*_.
*S*_*5*_: proportion of time spent in the full cubicle area (C_T_)	*S*_5_ = *a*_0_ + *a*_1_*L*	-62.88	***F* = 8.13** **(*p* = 0.011)**	*a*_0_ = 0.666 ***a***_**1**_ **= 0.056 (*p* = 0.011)**	*L* has a significant positive effect on *S*_*5*_. Heteroscedasticity present (NCV: *p* = 0.024; non-lame cows have higher variance, see Fig 3E).
*S*_*6*_: proportion of time spent in right cubicles (zone C_1_)	*S*_6_ = *a*_0_ + *a*_2_*P* + *a*_3_*D*	-21.32	***F* = 10.82** **(*p* < 0.001)**	*a*_0_ = 0.189 ***a***_**2**_ **= 0.073 (*p* < 0.001)** ***a***_**3**_ **= - 0.001 (*p* = 0.038)**	*P* has a significant positive effect on *S*_*6*_. *D* has a significant negative effect on *S*_*6*_.
*S*_*7*_: proportion of time spent in central cubicles (zone C_2_)	*S*_7_ = *a*_0_ (Intercept only)	-11.43	n/a	*a*_0_ = 0.400	No significance.
*S*_*8*_: proportion of time spent in left cubicles (zone C_3_)	*S*_8_ = *a*_0_ + *a*_2_*P*	-16.30	***F* = 9.65** **(*p* = 0.006)**	*a*_0_ = 0.537 ***a***_**2**_ **= - 0.068 (*p* = 0.006)**	*P* has a significant negative effect on *S*_*8*_.
*S*_*9*_: full range size (95% UD isopleth)	*S*_9_ = *a*_0_ + *a*_1_*L*	170.28	***F* = 5.66** **(*p* = 0.029)**	*a*_0_ = 150.84 ***a***_**1**_ **= - 15.88 (*p* = 0.029)**	*L* has a significant negative effect on *S*_*9*_.
*S*_*10*_: core range size (50% UD isopleth)	*S*_10_ = *a*_0_ + *a*_1_*L* + *a*_3_*D* + *a*_4_*L*:*D*	106.12	***F* = 7.24** **(*p* = 0.003)**	*a*_0_ = 11.531 *a*_1_ = 6.855 (*p* = 0.063) ***a***_**3**_ **= 0.02 (*p* = 0.002)** ***a***_**4**_ **= - 0.076 (*p* = 0.008)**	(*L* has a weak positive effect on *S*_*10*_). *D* has a significant positive effect on *S*_*10*_. Significant negative interaction effect between *L* and *D*.
*S*_*11*_: site fidelity (full upper barn & full range)	*S*_11_ = *a*_0_ (Intercept only)	-40.81	n/a	*a*_0_ = 0.472	No significance. Outlier cow (2596) removed to ensure normality of residuals (*n* = 19).
*S*_*12*_: site fidelity (feeding area & full range).	*S*_12_ = *a*_0_ + *a*_1_*L* + *a*_3_*D*	-42.69	***F* = 3.995 (*p* = 0.039)**	*a*_0_ = 0.688 ***a***_**1**_ **= -0.077 (*p* = 0.025)** ***a***_3_ = -0.0006 (*p* = 0.060)	*L* has a significant negative effect on *S*_*12*_. (*D* has a weak negative effect on *S*_*12*_). Outlier cow (2596) removed to ensure normality of residuals (*n* = 19).
*S*_*13*_: site fidelity (cubicle area & full range)	*S*_13_ = *a*_0_ + *a*_1_*L*	-28.26	***F* = 4.99** **(*p* = 0.039)**	*a*_0_ = 0.355 ***a***_**1**_ **= 0.102 (*p* = 0.039)**	*L* has a significant positive effect on *S*_*13*_. Outlier cow (2596) removed to ensure normality of residuals (*n* = 19).
*S*_*14*_: site fidelity (full upper barn & core range)	*S*_14_ = *a*_0_ + *a*_1_*L*	-25.32	***F* = 4.64** **(*p* = 0.046)**	*a*_0_ = 0.181 ***a***_**1**_ **= 0.106 (*p* = 0.046)**	*L* has a significant positive effect on *S*_*14*_. Outlier cow (2596) removed to ensure normality of residuals (*n* = 19). Heteroscedasticity present (NCV: *p* = 0.017; lame cows have higher variance, see Fig 4D).
*S*_*15*_: site fidelity (feeding area & core range)	*S*_15_ = *a*_0_ + *a*_1_*L*	-40.47	***F* = 10.69** **(*p* = 0.004)**	*a*_0_ = 0.295 ***a***_**1**_ **= - 0.112 (*p* = 0.004)**	*L* has a significant negative effect on *S*_*15*_.
*S*_*16*_: site fidelity (cubicle area & core range)	*S*_16_ = *a*_0_ + *a*_1_*L*	-34.37	***F* = 7.89** **(*p* = 0.013)**	*a*_0_ = 0.133 ***a***_**1**_ **= 0.106 (*p* = 0.013)**	*L* has a significant positive effect on *S*_*16*_. Outlier cows (2010 & 2596) removed to ensure normality of residuals (*n* = 18).

Results highlighted in bold indicate significance (*p* < 0.05). For all linear models considered, the intercept was always found to be significant and is always included. The Shapiro-Wilks test was used to test the normality of model residuals: for *S*_*11*_ *–S*_*14*_, a single outlier non-lame cow (2596) was removed to ensure normality; for *S*_*16*_, two outlier cows (2010, lame; 2596, non-lame) were removed to ensure normality. The non-constant variance (NCV) test was used to confirm the absence of heteroscedasticity in the model residuals (results non-significant, except for *S*_*5*_ and *S*_*14*_). AICc = Akaike Information Criterion score, corrected for small sample sizes. *L* = lameness (1 = lame, 0 = non-lame), *P* = parity, *D* = days in milk.

Parity was found to have a significant positive effect on the proportion of time spent in the right-hand cubicles, C_1_ (*S*_*6*_, p < 0.001; Fig 3F), and a significant negative effect on the proportion of time spent in the left-hand cubicles, C_3_ (*S*_*8*_, p = 0.006; Fig 3H). Given the relative locations of these cubicle zones (Fig 1), these results are entirely consistent with the fact that parity also had a significant positive effect on mean *x* location (*S*_*2*_, p < 0.001; Fig 3B); higher parity cows consistently spent more time in the area to the right-hand side of the upper barn.

Days in milk was found to have a significant negative effect on the proportion of time spent in the right-hand cubicles, C_1_ (*S*_*6*_, *p* = 0.038), although this effect was not as strong as the (positive) effect of parity within the same linear model (Fig 3F). Days in milk was also found to have a significant positive effect on the core (50% isopleth) range size, (*S*_*6*_, *p* = 0.002), although there was also a significant negative interaction effect with lameness (*p* = 0.008), see Fig 3J. This latter result can be interpreted as days in milk having a (strong) positive effect on core range size for non-lame cows and a (weaker) negative effect on core range size for lame cows (see respectively the green and red dashed lines in Fig 3J). However, this somewhat contradicts the finding that lameness (considered on its own within the same linear model) has a weak positive effect on core range size (*p* = 0.063). A complicated model interaction effect such as this should be interpreted with caution given the small sample size within our study.

None of the predictor variables were found to have any significant effects on the mean hourly walking distance (*S*_*1*_, Fig 3A), or the proportion of time spent in the central cubicle area, C_2_ (*S*_*7*_, Fig 3G).

### Site fidelity

**Fig 4:**
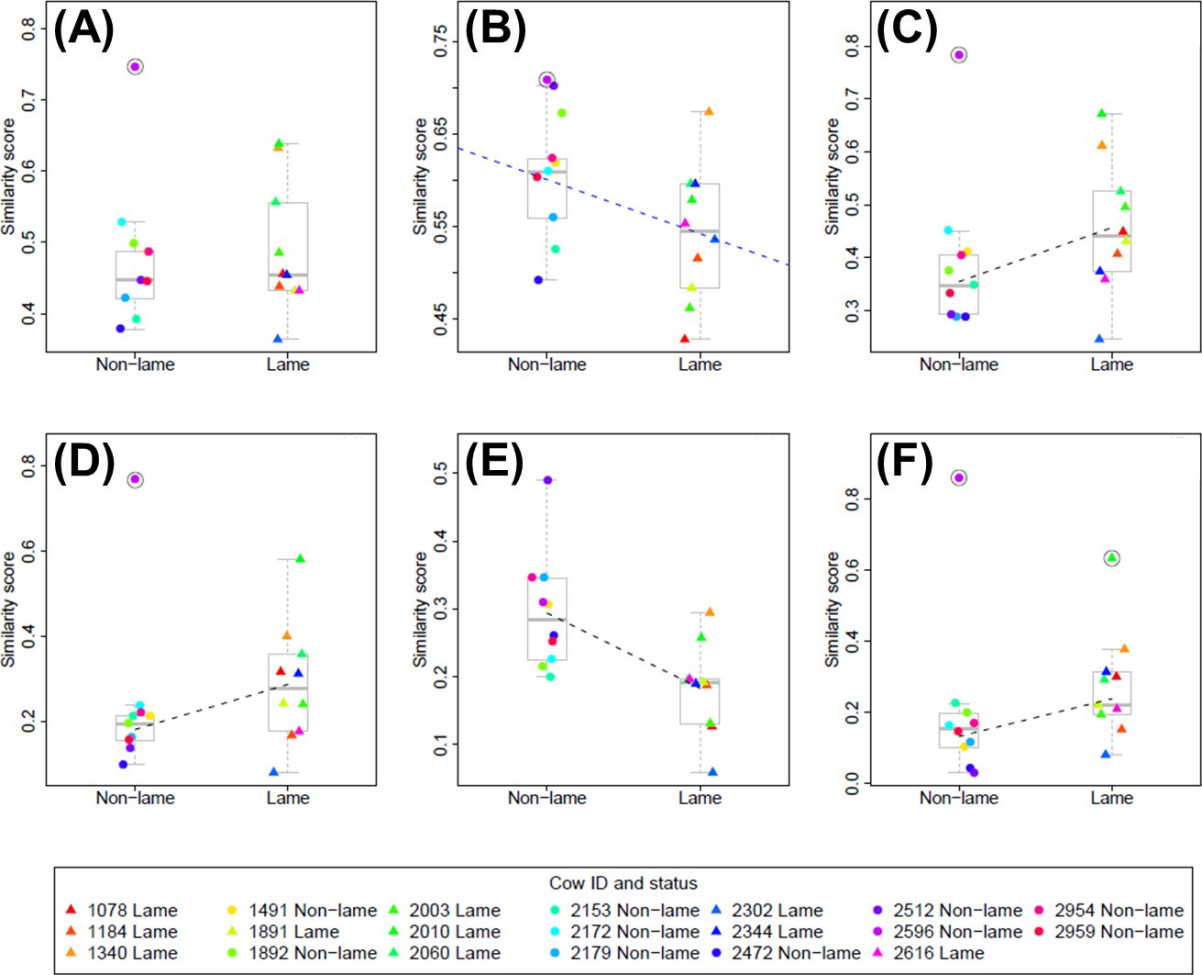
Plots showing relationship between significant predictor variables (lameness; parity; days in milk, DIM) and site-fidelity similarity measures. Data for each site-fidelity similarity measure, *S*_*11*_ to *S*_*16*_, are shown in plots (A) to (F) respectively, and are plotted against lameness status (which is the most significant predictor variable determined from the model selection procedure (Table 1), in all cases except (A), where no predictor variable is significant). Boxplots are used to show the spread of the data for the non-lame and lame groups, and individual data points are calculated as a mean average across all five days of the trial for each cow (S3 Table). Lame cows are plotted as filled triangles and non-lame cows as filled circles; the colours used to indicate each data point are fixed for each cow and are consistent across all plots (see legend). In (C-F), where the best fitting linear model includes only a single predictor variable, the fitted regression line is shown as a dashed black line. In (B) the best fitting linear model includes both lameness and DIM terms (Table 1); a regression line fitted only to the lameness variable (the most significant predictor) is shown as a blue dashed line for illustrative purposes only. In (A-D) and (F) the outlier cows (2596 and 2010) are marked with a black ring. Outlier cows were not included in the data for the purposes of model fitting (except for (E), where no outlier cows were removed from the data).

In general, site fidelity was higher for the full range (95% isopleth; Fig 4A–4C) than for the core range (50% isopleth; Fig 4D–4F); see S3 Table for site fidelity statistics for individual cows. Analysis of some site fidelity measures (*S*_*11*_ to *S*_*14*_) was strongly affected by a single outlier non-lame cow (2596), which resulted in the fitted model residuals being rejected as normal. Removal of this outlier cow (and also an additional lame outlier cow, 2010, for *S*_*16*_), led to the model residuals being accepted as normal, and results are presented on this basis. Given the reduced sample size, results with outlier(s) removed should be treated with caution. Only the fitted model for *S*_*15*_ resulted in normally distributed residuals without removal of outliers.

Lameness was the only predictor variable to have a significant effect on site fidelity (although days in milk had a weak negative effect on *S*_*12*_). Lame cows had significantly higher site fidelity than non-lame cows in the full cubicle area at both the full range (*S*_*13*_, *p* = 0.039; Fig 4C) and core range (*S*_*16*_, *p* = 0.013; Fig 4F), and also for the full upper barn area at the core range (*S*_*14*_, *p* = 0.046; Fig 4D). However, heteroscedasticity was present in the residuals for this latter result (lame cows had significantly higher variance in site fidelity). Non-lame cows had significantly higher site fidelity than lame cows in the feeding area at both the full range (*S*_*12*_, *p* = 0.025; Fig 4B) and core range (*S*_*15*_, *p* = 0.004; Fig 4E). It should be noted that this latter result is the only fitted model that satisfies the assumption of residual normality without removing outliers from the data, and hence can be considered more robust.

### Predictive model for lameness

Table 2 illustrates that the predictive model structure with the lowest AIC score, and hence the best relative fitting model (accounting for model complexity), is of the form

$$log\left(\frac{p}{1-p}\right)=b_{0}+b_{4}S_{4}+b_{9}S_{9},$$ (5)

where *S*_*4*_ is the proportion of time spent in the feeding area, F, and *S*_*9*_ is the number of cells in the full range (95% isopleth). This model correctly predicts the lameness status of 18 out of the 20 cows within the study (S4 Table). Other model structures that include one or more of the mean *x* coordinate (*S*_*2*_), the proportion of time spent in the full cubicle area (*S*_*5*_), or the site fidelity in the feeding area (core range, *S*_*15*_), are also able to correctly identify the lameness status of at least 18 out of the 20 cows, although these models have a worse AICc score due to having more complex structures with additional parameters. Across all the best-fitting models in Table 2, cow 2153 (non-lame) is always incorrectly classified as lame. However, investigation of the health records for this cow revealed that it may have been misclassified by the expert observers at the start of the study (see Discussion), and hence the models are all essentially correct in this case.

**Table 2:** Best fitting model structures considered for logistic regression predictive model with associated Akaike Information Criterion (AICc) scores (corrected for small sample sizes).

AICc score	Model structure & regression coefficients	Correct predictions	Incorrectly predicted cow IDS
20.21	*b*_0_ + *b*_4_*S*_4_ +*b*_9_*S*_9_ *b*_0_ = 25.61, *b*_4_ = -39.36, *b*_9_ = -0.098	18/20	2153, 2344
20.27	*b*_0_ + *b*_4_*S*_4_ +*b*_5_*S*_5_ + *b*_9_*S*_9_ *b*_0_ = 153.29, *b*_4_ = -161.70, *b*_5_ = -120.02, *b*_9_ = -0.158	18/20	2153, 2344
21.50	*b*_0_ + *b*_4_*S*_4_ +*b*_15_*S*_15_ *b*_0_ = 12.64, *b*_4_ = -23.96, *b*_15_ = -23.46	18/20	1340, 2153
21.88	*b*_0_ + *b*_2_*S*_2_ +*b*_4_*S*_4_ + *b*_15_*S*_15_ *b*_0_ = 4.67, *b*_2_ = 0.221, *b*_4_ = -19.08, *b*_15_ = -24.35	19/20	2153
21.98	*b*_0_ + *b*_4_*S*_4_ +*b*_9_*S*_9_ + *b*_15_*S*_15_ *b*_0_ = 24.33, *b*_4_ = -30.10, *b*_9_ = -0.080, *b*_15_ = -16.62	18/20	2010, 2153

AICc scores are listed in ascending order with lower values corresponding to a better relative model fit. The model is fitted through a logit link function for the lameness binary variable (0 = non-lame, 1 = lame). All models include an intercept. The dependent variables considered in the model selection are those found to be significant in the statistical analysis shown in Table 1 and are given by: *S*_*2*_: mean *x* coordinate; *S*_*4*_: proportion of time spent in the feeding area (F); *S*_*5*_: proportion of time spent in the full cubicle area (C_T_); *S*_*9*_: full range size (95% UD isopleth); *S*_*15*_: site fidelity (feeding area & core range). All other model structures considered had higher AICc scores (AICc > 22) and are not shown.

## Discussion

By collecting high resolution spatial location data we have demonstrated in this 5-day cross-sectional study how groups of 10 lame and 10 non-lame cows exhibit a number of statistically significant differences in their movement and space-use behaviour (Table 1), including level of site fidelity, range size, and time spent in specific locations of the barn. Furthermore, we have shown that only two of these space-use measures need to be included within a simple statistical model in order to accurately predict the lameness status of all individual cows within the herd (S4 Table). Lameness is one of the key health and welfare issues affecting dairy cattle globally [3]. Early detection of lameness can reduce animal pain and suffering [5], and also minimise potential costs to farmers [4]. Current lameness detection methods, usually based on expert observations of mobility, can be time-intensive [6] and hence there is a need for novel automated methods of detection. We have demonstrated in this study how a RTLS wireless local positioning system can be used to continuously monitor movement and space-use behaviour at high recording frequency, providing additional sources of behavioural information that cannot be easily collected using other systems based on accelerometers or video [7,8]. This type of RTLS space-use monitoring system could potentially be extended within a Precision Livestock Farming approach [9] to enable automated on-farm prediction of lameness status in individual cows based on space-use and other behavioural differences.

Our finding that non-lame cows spend a higher proportion of their time in the feeding area (*S*_*4*_, Table 1; Fig 3D), and the equivalent result that lame cows spend more time in the cubicles area (*S*_*5*_, Table 1; Fig 3E), is consistent with existing studies on feeding behaviour in dairy cows [26–28,30]. In this study we do not try to distinguish between cows observed in the feeding area that are actually feeding and those that are not feeding. However, this distinction may be possible by combining basic spatial location data with additional accelerometer data on activity [28]. Although we didn’t measure feed intake directly in this study, earlier studies have shown that lame cows may eat the same amount but at a faster rate than non-lame cows [26]. This may reflect a reduced time spent at the feed face in order to avoid confrontation and competition from other cows, since lame cows are known to be less likely to start an aggressive interaction [10]. Lame cows may also increase their time spent lying [31] in order to reduce discomfort and pain [1], and this could also explain our observed results.

Non-lame cows had significantly higher site fidelity than lame cows in the feeding area (*S*_*12*_ and *S*_*15*_, Table 1; Fig 4B and 4E), and this result holds at the core range even with the outlier cow (2596) included in the analysis. Non-lame cows could be more able, or choosing, to compete for their preferred food locations and consistently revisit these areas, whereas lame cows may be avoiding potential competition and confrontation at the feed face [10]. The spacing of dairy cows at a food trough is known to depend on dominance rank at small group sizes [70], and both dairy cows and buffalo cows are known to show preferences for specific sites within the milking parlour [71,72]. In wild animals, high levels of site fidelity in foraging locations have been observed, albeit with high individual variance related to underlying environmental conditions or prey availability [20]. When the outlier non-lame cow (2596) is removed from the analysis then lame cows are found to have significantly higher site fidelity than non-lame cows in the upper barn area for their core range and also for the cubicles area at both their full range and core range (*S*_*13*_, *S*_*15*_, and *S*_*16*_, Table 1; Fig 4C, 4E and 4F). This indicates that lame cows are more likely than non-lame cows to return to the same location within the cubicles area on a day-to-day basis. It should be noted that investigation of farm health records for cow 2596 showed no evidence of any serious underlying health issues or related treatments before or after the study period, and with this cow included in the analysis, the results are no longer significant and the linear model is not valid (due to non-normality of residuals). The apparent pattern of higher site fidelity shown in these areas by lame cows (Fig 4) should be investigated further in future studies with larger sample sizes.

Non-lame cows had a significantly larger full range size than lame cows (*S*_*9*_, Table 1; Fig 3I), even though there was no difference in total walking distance between the two groups (*S*_*1*_, Table 1; Fig 3A). In contrast, the core range size was (weakly) positively influenced by lameness, and by the number of days in milk (*S*_*10*_, Table 1), with a negative interaction term between the two predictor variables (Fig 3J). However, the complexity of this model means it should be treated with some scepticism given the small sample sizes in the study (the complex model structure could potentially be due to the influence of a small number of specific individual cows). Additionally, although the sizes of the full and core ranges for each individual cow are an important measure of how they use the space available within the barn, they may not capture all relevant features of their behaviour; areas visited very infrequently may still be biologically important (e.g. visits to the water trough or brush may be infrequent, but still play an important role in the daily activity of each cow).

Parity was found to have a strong effect on the horizontal (mean *x*) location within the barn (*S*_*2*_, p < 0.001; Fig 3B), with higher parity cows spending more time in the right-hand cubicles, C_1_ (*S*_*6*_, p < 0.001; Fig 3F), and lower parity cows spending more time in the left-hand cubicles, C_3_ (*S*_*8*_, p = 0.006; Fig 3H). The right-hand side of the barn used in our study corresponds to being close to the passageway to the milking parlour (Fig 1), and hence the difference in horizontal location could be because older and more experienced (higher parity) cows are choosing to stay near the connecting passage to the milking parlour in order to get a better position in the milking queue. Disease status is also known to affect milking order, with lame cows more likely to be found in the last third of the milking [29,73] and taking longer to return from the milking parlour [11]. Similarly, cows suffering from mastitis were found to enter the milking parlour later [74], although the same study reported no effect of age, parity or days in milk on milking order. An alternative interpretation of our results is that when returning from milking, the older higher parity cows in our study are simply not spreading out within the barn as much as younger cows, possibly because they have longer bouts of low activity (standing or lying) and spend less time feeding. For example, previous studies have reported that primiparous (parity 1) cows have significantly more lying bouts of shorter duration when compared to parity 2 and parity 3+ groups [75] and that parity 1 and 2 cows spend more time feeding than parity 3+ cows [76]. Higher parities have also been associated with longer standing times [77]. It is also possible that there is a social aspect to this observed space-use behaviour, with cows of similar parity staying close to each other in different areas of the barn for social reasons. Other potential factors such as localised air quality, temperature, wind, and noise may also influence the preferential use of certain locations within the barn by individual cows, but were variables that were not measured in this study.

Days in milk (DIM) was found to have a significant negative effect on the proportion of time spent in the right-hand cubicles, C_1_ (*S*_*6*_, *p* = 0.038) and a significant positive effect on the core (50% isopleth) range size, (*S*_*6*_, *p* = 0.002) with an associated negative interaction effect with lameness (Fig 3J). Various studies have reported increased lying behaviour with increased DIM [78,79], while increased DIM has also been shown to lead to decreased feeding frequency but increased meal duration and total feeding time [76]. The interplay between DIM, parity and lameness is clearly complex, and further studies are needed to explore how observed space-use behaviour is driven by each of these factors and their potential interactions.

Although we have high resolution spatial location data for each individual cow, we also have relatively small sample sizes (10 lame and 10 non-lame cows) and the cross-sectional study ran for only 5 days. Hence, although our results have exciting potential, we are cautious about over-generalisation. In particular, the model parameter values found during the statistical analysis are specific to this study group and barn environment and will almost certainly be different for other cows or other barn locations. We have demonstrated how space-use measures in individual cows are linked to health (lameness) status, parity, and (to a lesser extent) days in milk, but space-use behaviour is also likely to be influenced by management actions, the barn landscape and layout, the frequency of milking and the milking system used (automated v milking parlour), and individual cow age and breed [8]. Similar to [8], as we have undertaken a short-term cross-sectional study using cows with known lameness status, it is not possible to determine from our results how well space-use behavioural indicators may perform in detecting changes in the status of individual cows as they transition from non-lame to lame (and subsequently recover after treatment) over the longer term. Longitudinal studies over an extended time period with larger group sizes would allow us to determine the consistency of any observed space-use differences, as well as what space-use behaviour changes might be detectable at the onset of lameness. By monitoring a full herd across a larger time period it would also be possible to determine more detailed social interactions and spatial dynamics that may influence individual space-use behaviour. In this study, the cows being tracked formed a subset of a much larger herd, and we did not attempt to explore social interactions because of the difficulty in distinguishing between direct and indirect social interactions when many individuals within the full herd are not part of the observed data set. Nevertheless, our results suggesting higher parity cows use different areas of the barn compared to lower parity cows (Fig 3F and 3H) hints at a possible social aspect to their space-use behaviour. More detailed analysis of social behaviour could be undertaken by exploring network features within the herd as a whole [80], or through pairwise analyses of space-use and space-use similarity [23,49,63,64,81].

Our aim with the predictive model in Eq (4) is to illustrate the ‘proof of concept’ of how observed space-use behavioural data can be used to give an accurate prediction of lameness status in individual cows in this cross-sectional study. As it stands, the model is not directly transferable to other groups of cows or barn locations and would need to be adapted and tested before being used in other farm environments. Nevertheless, it demonstrates the principle of how only a few simple space-use measures could be used to accurately determine lameness status for individual cows within a herd. The best relative fitting model structure only included time spent in the feeding area, and the number of virtual cells in the full range (Table 2), demonstrating that as few as two simple space-use measures are needed to give a good description of lameness status in this study group of cows. Such a simple predictive model could potentially be quickly adapted and parameterised for practical on-farm use (assuming the general results hold), unlike more complex predictive models that might require computationally intensive model fitting or continuous re-parametrisation.

Out of 20 cows, only one lame cow (2344) and one non-lame cow (2153) were incorrectly classified by the best-fitting predictive model (Table 2, S4 Table). Investigation of the health records of cow 2153 suggests that she was likely to have been misclassified as ‘non-lame’ before the study by the expert observers (through mobility scoring), as lesions with the potential to cause lameness were found on her feet when all cows were inspected at the end of the study period (and hence the model prediction was essentially correct, and was able to detect this earlier misclassification by the expert observers). In March 2014 shortly after our study was completed this cow underwent a series of 11 treatments for mastitis and was eventually culled early. Mastitis is also known to affect dairy cow behaviour, with reduced lying times, reduced feed intake and a reduction in competitive behaviour at the feeder compared to healthy cows [32,82]. No other cows from the non-lame trial group had treatments for any health conditions during the study period (or for at least 3 months after the study had finished). Meanwhile, when inspected at the end of the study period, cow 2344 (lame) was found to be wearing a hoof block, which is fitted to relieve pressure on the affected areas of the hoof, and hence this may have potentially reduced clinical signs and changes in behaviour related to lameness for this cow. No other cows in the lame group had similar treatments during the study period (or for at least 3 months after the study had finished).

Increasing demand for animal products and intensification of farming practices in general, means that there is a need for automated behavioural monitoring systems that can act as an ‘early warning’ to detect and predict the health status of managed animals, including dairy cows suffering from lameness and other diseases [7,8,26,83]. Automated lameness detection technology systems have been developed based on the identification of an abnormality of gait or posture [83], using force plate technology [7,84] or kinematics [85]. Meanwhile, automated monitoring of feeding behaviour in cattle has relied on electronic feed troughs [27,30]. However, there has not been a widespread uptake of such systems on commercial farms due to the high price, practical limitations such as lack of space, or limited precision of detection [7]. Automated lameness detection systems based on differences in locomotion or activity patterns observed in accelerometer data have been suggested as a lower cost alternative approach [7,8,35]. The results we present here suggest that space-use and site-fidelity measures could be an exciting addition to the suite of behavioural indicators available as part of Precision Livestock Farming approaches for monitoring and detecting diseases such as lameness in cattle and other animals.

The use of space-use and site-fidelity measures as health status indicators does not need to be limited to cattle or dairy cows, and similar approaches could also potentially be used with other managed animal species or even wild animals, if similar differences in space-use behaviour linked to health status are found to exist. Little is known about the direct link between space-use behaviour and health in pigs, although there is evidence suggesting that impoverished environments contribute to high levels of boredom and apathy [86]. It should be straightforward to monitor space-use patterns of individual pigs using automated wireless positioning system in a similar manner to what we have done in this study with dairy cows. In the context of broiler chickens, [87] showed how optical flow, a measure of the movement and flow of the flock as a whole through the space within the barn determined by computer vision techniques, could be directly linked to the health and disease status of the flock, illustrating how space-use metrics at the group-level can also be used as indicators for welfare monitoring.

## Conclusions

We have demonstrated in this study how location tracking data collected from animal-mounted wireless sensors using a Real Time Location System can be processed and analysed to give a suite of space-use behavioural measures. We have used these measures to explore differences in space-use behaviour in two test groups of barn-housed dairy cows in a cross-sectional study design, and found significant differences between lame and non-lame individuals. Non-lame cows had higher site fidelity, and spent more time, in the feeding area, and had a larger range within the barn. In contrast, lame cows spent less time in the feeding area and more time in the cubicle areas of the barn, where they had higher site fidelity. Space-use behaviour was also found to be influenced by parity and days in milk: higher parity cows had a mean location closer to where the connecting passage to the milking parlour is situated, and days in milk was found to influence the core range size. We have demonstrated that only two simple space-use measures, proportion of time spent in the feeding area and full range size, are needed within a simple statistical model in order to accurately predict the lameness status of all individual cows within the herd. The sample size used within this study (10 lame and 10 non-lame cows) was small and hence care should be taken in directly extrapolating our results and conclusions to other studies and contexts. However, the general findings and associated methods for exploring animal space-use could potentially be developed in future studies to form a new set of tools for automated monitoring of dairy cattle, or for monitoring, detecting and predicting health status in other managed or wild animal species.

## Supporting information

S1 Table Health and milk production data for cows used within the study. (DOCX)

S2 Table Summary space-use statistics for each cow within the study. (DOCX)

S3 Table Summary site-fidelity statistics for each cow within the study. (DOCX)

S4 Table True and predicted probability of lameness for each cow in the study using best relative fitting predictive model. (DOCX)

S1 File Space-use intensity plots for all cows and all study days. S1 File contains space-use intensity plots (UDs) for all cows over all five days of the trial. The space-use intensity UD is calculated by overlaying a 1.5m x 1.5m square grid (40 x 13 cells) onto the upper barn area only and counting the cells in which the smoothed trajectory points for each cow occur for each day of the trial. Darker colours correspond to higher space-use intensity. The 95% and 50% isopleths (corresponding to the full and core ranges for movement within the upper barn area only) are respectively indicated by the dashed and solid contour lines.
(PDF)

S2 File Basic space-use measures for each study day. S2 File contains box-plots showing basic space-use measures by day of the trial. Lame cows are marked using triangles and non-lame cows are marked using circles. The colours used to indicate each cow are consistent across all plots. There are no clear trends by day in any of the basic space-use measures considered.
(PDF)

S3 File Location data for all cows and study days. S3 File contains the raw location tracking data for each cow for each day of the study as used in the analysis.
(CSV)
