# Supplementary material for: Space-use patterns highlight behavioural differences linked to lameness, parity, and days in milk in barn-housed dairy cows
Source: PLoS One. 2018 Dec 19;13(12):e0208424. doi: 10.1371/journal.pone.0208424 (PMC6300209; doi:10.1371/journal.pone.0208424)
Supplement: S1 Table — (DOCX) [file pone.0208424.s001.docx]

**S1 Table. Health and milk production data for cows used within the study.**

| **Cow ID** | **Lameness status^a^** | **Parity** | **Days in Milk (DIM)^b^** | **Mean Daily Yield^b^** |
| --- | --- | --- | --- | --- |
| 1078 | 1 | 6 | 220 | 43.7 |
| 1184 | 1 | 6 | 150 | 48.7 |
| 1340 | 1 | 6 | 44 | 31.1 |
| 1491 | 0 | 4 | 174 | 43.3 |
| 1891 | 1 | 4 | 45 | 44.9 |
| 1892 | 0 | 4 | 107 | 45.2 |
| 2003 | 1 | 4 | 73 | 47.8 |
| 2010 | 1 | 3 | 144 | 43.6 |
| 2060 | 1 | 3 | 136 | 58.4 |
| 2153 | 0 | 3 | 111 | 48.6 |
| 2172 | 0 | 3 | 114 | 45.2 |
| 2179 | 0 | 3 | 218 | 41.1 |
| 2302 | 1 | 3 | 154 | 41.6 |
| 2344 | 1 | 3 | 45 | 44.2 |
| 2472 | 0 | 2 | 175 | 43.5 |
| 2512 | 0 | 2 | 99 | 43.3 |
| 2596 | 0 | 2 | 78 | 32.9 |
| 2616 | 1 | 2 | 118 | 43.6 |
| 2954 | 0 | 1 | 117 | 30.4 |
| 2959 | 0 | 1 | 174 | 28.7 |

^a^Lameness status: 1 = lame; 0 = non-lame (based on mobility scoring prior to study).

^b^Days in Milk and Mean Daily Yield were calculated for the current parturition only.
