# Supplementary material for: Space-use patterns highlight behavioural differences linked to lameness, parity, and days in milk in barn-housed dairy cows
Source: PLoS One. 2018 Dec 19;13(12):e0208424. doi: 10.1371/journal.pone.0208424 (PMC6300209; doi:10.1371/journal.pone.0208424)
Supplement: S2 Table — (DOCX) [file pone.0208424.s002.docx]

**S2 Table. Summary space-use statistics for each cow within the study.**

| **Cow ID** | ***S_1_*: mean distance moved per hour (m)** | ***S_2_*: mean *x* co-ordinate (m)** | ***S_3_*: mean *y* co-ordinate**  **(m)** | ***S_4_*: proportion of time spent in the feeding area (F)** | ***S_5_*: proportion of time spent in the full cubicle area (C_T_)** | ***S_6_*: proportion of time spent in right cubicles (zone C_1_)** | ***S_7_*: proportion of time spent in central cubicles (zone C_2_)** | ***S_8_*: proportion of time spent in left cubicles (zone C_3_)** | ***S_9_*: full range size (95% UD isopleth)** | ***S_10_*: core range size (50% UD isopleth)** |
| --- | --- | --- | --- | --- | --- | --- | --- | --- | --- | --- |
| 1078 | 117.6 | 33.54 | 24.28 | 0.2209 | 0.7517 | 0.2558 | 0.6314 | 0.1128 | 141.4 | 18 |
| 1184 | 115.0 | 35.90 | 22.94 | 0.2490 | 0.7410 | 0.4468 | 0.4344 | 0.1188 | 131.4 | 15.6 |
| 1340 | 106.2 | 38.71 | 23.14 | 0.2761 | 0.7114 | 0.6674 | 0.1003 | 0.2323 | 127.2 | 19.4 |
| 1491 | 100.8 | 30.64 | 22.49 | 0.3050 | 0.6889 | 0.3501 | 0.4230 | 0.2269 | 146.4 | 23.2 |
| 1891 | 96.0 | 37.43 | 23.58 | 0.3090 | 0.6818 | 0.5391 | 0.3663 | 0.0946 | 111.6 | 13.4 |
| 1892 | 101.6 | 35.21 | 21.99 | 0.3978 | 0.5625 | 0.4893 | 0.2453 | 0.2654 | 140.4 | 21.2 |
| 2003 | 115.8 | 29.42 | 25.06 | 0.2442 | 0.7389 | 0.1500 | 0.7578 | 0.0923 | 123.4 | 20 |
| 2010 | 133.6 | 31.97 | 23.55 | 0.2381 | 0.7182 | 0.2323 | 0.5891 | 0.1787 | 164.4 | 20 |
| 2060 | 108.6 | 31.87 | 22.29 | 0.2598 | 0.7342 | 0.3510 | 0.3938 | 0.2553 | 144.2 | 20.4 |
| 2153 | 129.4 | 31.66 | 23.40 | 0.2082 | 0.7849 | 0.4388 | 0.2696 | 0.2916 | 150.2 | 19.4 |
| 2172 | 111.6 | 16.95 | 21.86 | 0.3632 | 0.6346 | 0.0527 | 0.2812 | 0.6662 | 141.2 | 19.6 |
| 2179 | 134.0 | 26.22 | 24.94 | 0.3249 | 0.6689 | 0.2156 | 0.4308 | 0.3536 | 150.8 | 26.2 |
| 2302 | 109.4 | 22.71 | 25.04 | 0.2487 | 0.7452 | 0.2700 | 0.2222 | 0.5077 | 125.2 | 15.6 |
| 2344 | 116.4 | 27.53 | 22.50 | 0.2906 | 0.6926 | 0.3051 | 0.1915 | 0.5034 | 153.0 | 20 |
| 2472 | 111.6 | 19.21 | 22.94 | 0.3531 | 0.6447 | 0.0775 | 0.3072 | 0.6154 | 143.0 | 23.2 |
| 2512 | 120.4 | 27.30 | 22.76 | 0.3179 | 0.6729 | 0.2882 | 0.3295 | 0.3823 | 161.4 | 23.4 |
| 2596 | 127.6 | 23.51 | 23.60 | 0.2810 | 0.7058 | 0.1129 | 0.6382 | 0.2489 | 177.8 | 13.2 |
| 2616 | 110.2 | 27.93 | 24.57 | 0.2855 | 0.7096 | 0.1971 | 0.4679 | 0.3351 | 127.8 | 19.2 |
| 2954 | 107.0 | 24.41 | 22.26 | 0.3649 | 0.6308 | 0.1589 | 0.4634 | 0.3777 | 130.4 | 18.4 |
| 2959 | 117.0 | 20.98 | 22.76 | 0.3255 | 0.6707 | 0.0634 | 0.4509 | 0.4857 | 166.8 | 28 |

All data are calculated as the daily mean value over the 5 days of the study and are displayed to 4 significant figures where appropriate.
