# Supplementary material for: Space-use patterns highlight behavioural differences linked to lameness, parity, and days in milk in barn-housed dairy cows
Source: PLoS One. 2018 Dec 19;13(12):e0208424. doi: 10.1371/journal.pone.0208424 (PMC6300209; doi:10.1371/journal.pone.0208424)
Supplement: S3 Table — (DOCX) [file pone.0208424.s003.docx]

**S3 Table. Summary site-fidelity statistics for each cow within the study.**

| **Cow ID** | ***S_11_*: site fidelity (full upper barn & full range)** | ***S_12_*: site fidelity (feeding area & full range).** | ***S_13_*: site fidelity (cubicle area & full range)** | ***S_14_*: site fidelity (full upper barn & core range)** | ***S_15_*: site fidelity (feeding area & core range)** | ***S_16_*: site fidelity (cubicle area & core range)** |
| --- | --- | --- | --- | --- | --- | --- |
| 1078 | 0.4555 | 0.4281 | 0.4493 | 0.3165 | 0.1266 | 0.3009 |
| 1184 | 0.4382 | 0.5157 | 0.4072 | 0.1672 | 0.1872 | 0.1523 |
| 1340 | 0.6331 | 0.6735 | 0.6117 | 0.4002 | 0.2944 | 0.3782 |
| 1491 | 0.4871 | 0.6181 | 0.4103 | 0.2120 | 0.3066 | 0.1026 |
| 1891 | 0.4318 | 0.4839 | 0.4313 | 0.2423 | 0.1921 | 0.2215 |
| 1892 | 0.4987 | 0.6727 | 0.3763 | 0.1947 | 0.2154 | 0.1985 |
| 2003 | 0.4855 | 0.4622 | 0.4957 | 0.2403 | 0.1311 | 0.1947 |
| 2010 | 0.6385 | 0.5788 | 0.6716 | 0.5813 | 0.2574 | 0.6349 |
| 2060 | 0.5565 | 0.5963 | 0.5251 | 0.3578 | 0.1942 | 0.2926 |
| 2153 | 0.3921 | 0.5258 | 0.3479 | 0.2119 | 0.1998 | 0.2250 |
| 2172 | 0.5285 | 0.6095 | 0.4507 | 0.2383 | 0.2250 | 0.1623 |
| 2179 | 0.4210 | 0.5594 | 0.2875 | 0.1610 | 0.3457 | 0.1177 |
| 2302 | 0.3636 | 0.5359 | 0.2458 | 0.0795 | 0.0586 | 0.0812 |
| 2344 | 0.4544 | 0.5960 | 0.3743 | 0.3121 | 0.1895 | 0.3147 |
| 2472 | 0.3782 | 0.4923 | 0.2892 | 0.0982 | 0.2613 | 0.0443 |
| 2512 | 0.4470 | 0.7019 | 0.2939 | 0.1357 | 0.4896 | 0.0312 |
| 2596 | 0.7463 | 0.7085 | 0.7817 | 0.7681 | 0.3100 | 0.8610 |
| 2616 | 0.4321 | 0.5533 | 0.3593 | 0.1761 | 0.1966 | 0.2099 |
| 2954 | 0.4865 | 0.6233 | 0.4044 | 0.2205 | 0.3460 | 0.1684 |
| 2959 | 0.4456 | 0.6036 | 0.3339 | 0.1553 | 0.2525 | 0.1456 |

All data are calculated as the daily mean value over the 5 days of the study and are displayed to 4 significant figures where appropriate.
