# Supplementary material for: Space-use patterns highlight behavioural differences linked to lameness, parity, and days in milk in barn-housed dairy cows
Source: PLoS One. 2018 Dec 19;13(12):e0208424. doi: 10.1371/journal.pone.0208424 (PMC6300209; doi:10.1371/journal.pone.0208424)
Supplement: S4 Table — (DOCX) [file pone.0208424.s004.docx]

**S4 Table**. **True and predicted probability of lameness for each cow in the study using best relative fitting predictive model.**

| **Cow ID** | **True lameness status**  **(1 = lame, 0 = non-lame)** | **Probability of lameness from model, *p***  **(2 decimal places)** | **Prediction correct** |
| --- | --- | --- | --- |
| 1078 | 1 | 0.95 | Y |
| 1184 | 1 | 0.95 | Y |
| 1340 | 1 | 0.90 | Y |
| 1891 | 1 | 0.92 | Y |
| 2003 | 1 | 0.98 | Y |
| 2010 | 1 | 0.52 | Y |
| 2060 | 1 | 0.77 | Y |
| 2302 | 1 | 0.97 | Y |
| 2344 | 1 | 0.30 | N |
| 2616 | 1 | 0.86 | Y |
| 1491 | 0 | 0.32 | Y |
| 1892 | 0 | 0.02 | Y |
| 2153 | 0 | 0.93 | N |
| 2172 | 0 | 0.07 | Y |
| 2179 | 0 | 0.12 | Y |
| 2472 | 0 | 0.09 | Y |
| 2512 | 0 | 0.06 | Y |
| 2596 | 0 | 0.05 | Y |
| 2954 | 0 | 0.17 | Y |
| 2959 | 0 | 0.03 | Y |

The true lameness status corresponds to the classification given to each cow at the start of the study by an expert observer using mobility scoring (S1 Table). The probability of lameness is determined from the best fitting logistic regression model (Equation (5), Table 2). As lameness status is binary, we assume that if *p* > 0.5 the model has predicted the cow to be lame, while if *p* < 0.5 the model has predicted the cow to be non-lame. Under this assumption, 18 of the 20 cows have their lameness status correctly predicted by the model. The two cows incorrectly classified are highlighted with a grey background in the table.
