# Supplementary material for: Space-use patterns highlight behavioural differences linked to lameness, parity, and days in milk in barn-housed dairy cows
Source: PLoS One. 2018 Dec 19;13(12):e0208424. doi: 10.1371/journal.pone.0208424 (PMC6300209; doi:10.1371/journal.pone.0208424)
Supplement: S1 File — contains space-use intensity plots (UDs) for all cows over all five days of the trial. The space-use intensity UD is calculated by overlaying a 1.5m x 1.5m square grid (40 x 13 cells) onto the upper barn area only and counting the cells in which the smoothed trajectory points for each cow occur for each day of the trial. Darker colours correspond to higher space-use intensity. The 95% and 50% isopleths (corresponding to the full and core ranges for movement within the upper barn area only) are respectively indicated by the dashed and solid contour lines. (PDF) [file pone.0208424.s005.pdf]

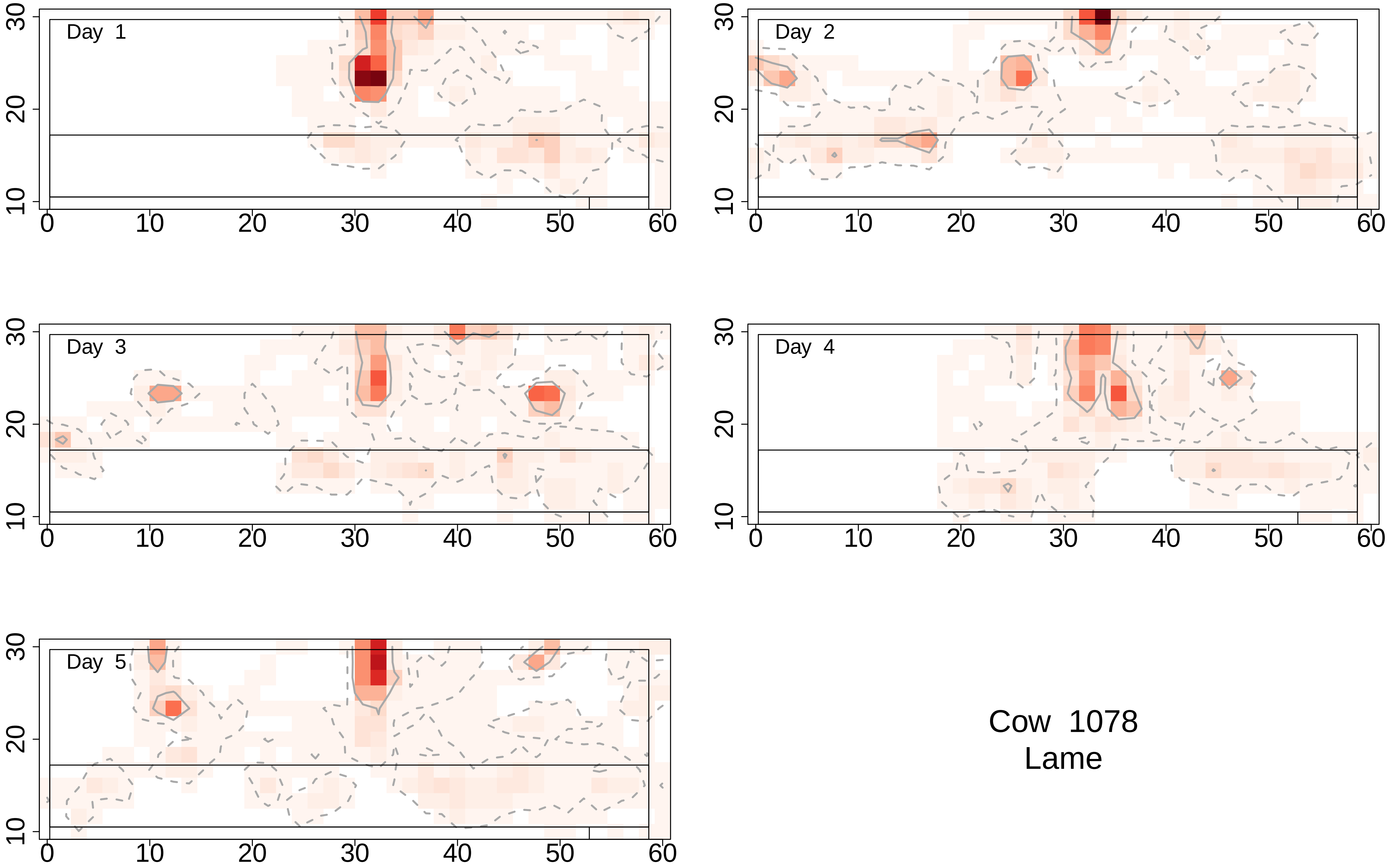
Day 1
Day 2
Day 3
Day 4
Day 5
Cow 1078
Lame

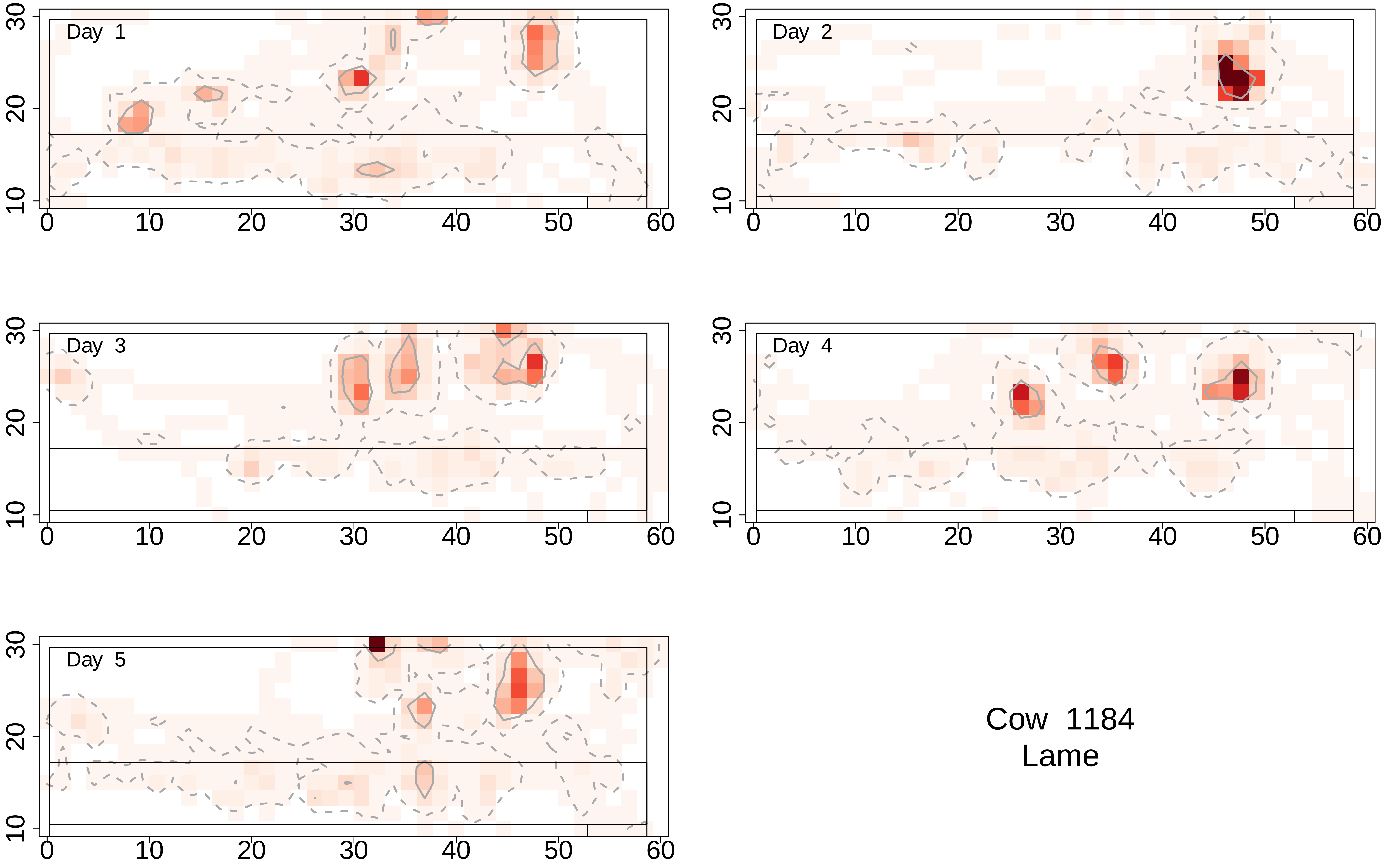
Day 1
Day 2
Day 3
Day 4
Day 5
Cow 1184
Lame

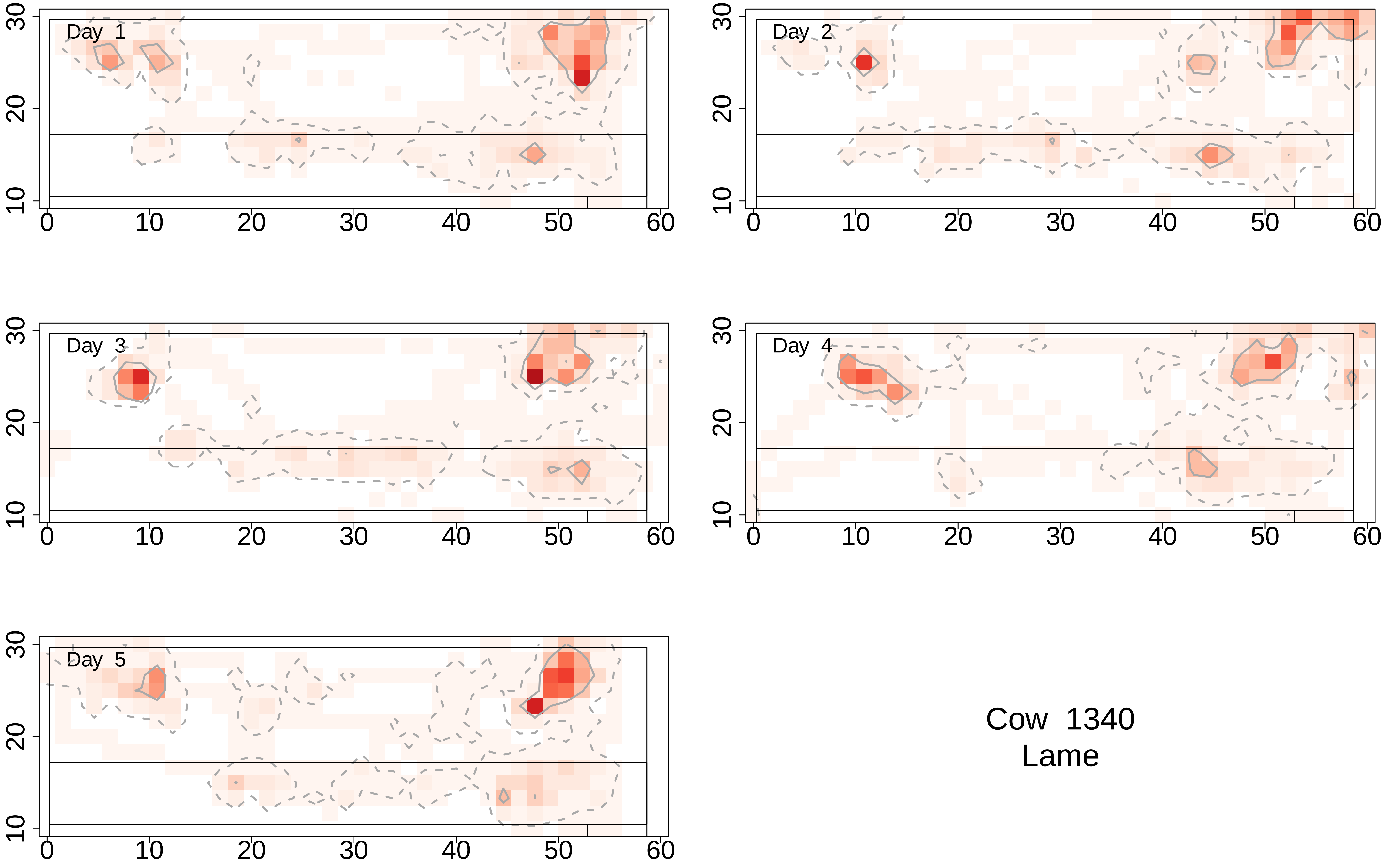
Day 1
Day 2
Day 3
Day 4
Day 5
10
20
30
0
10
20
30
40
50
60
Cow 1340
Lame

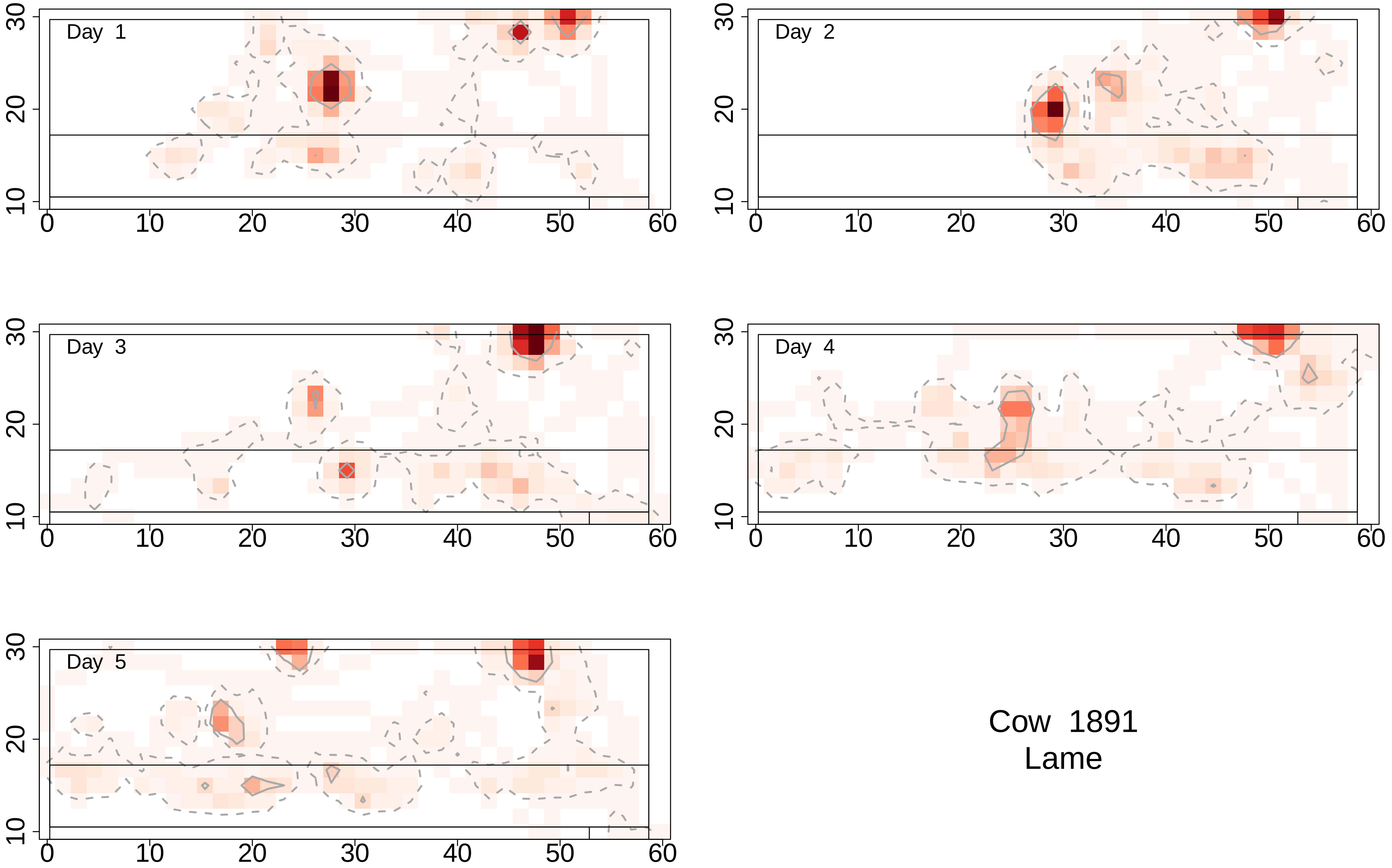

Day 1
Day 2
Day 3
Day 4
Day 5
Cow 1891
Lame

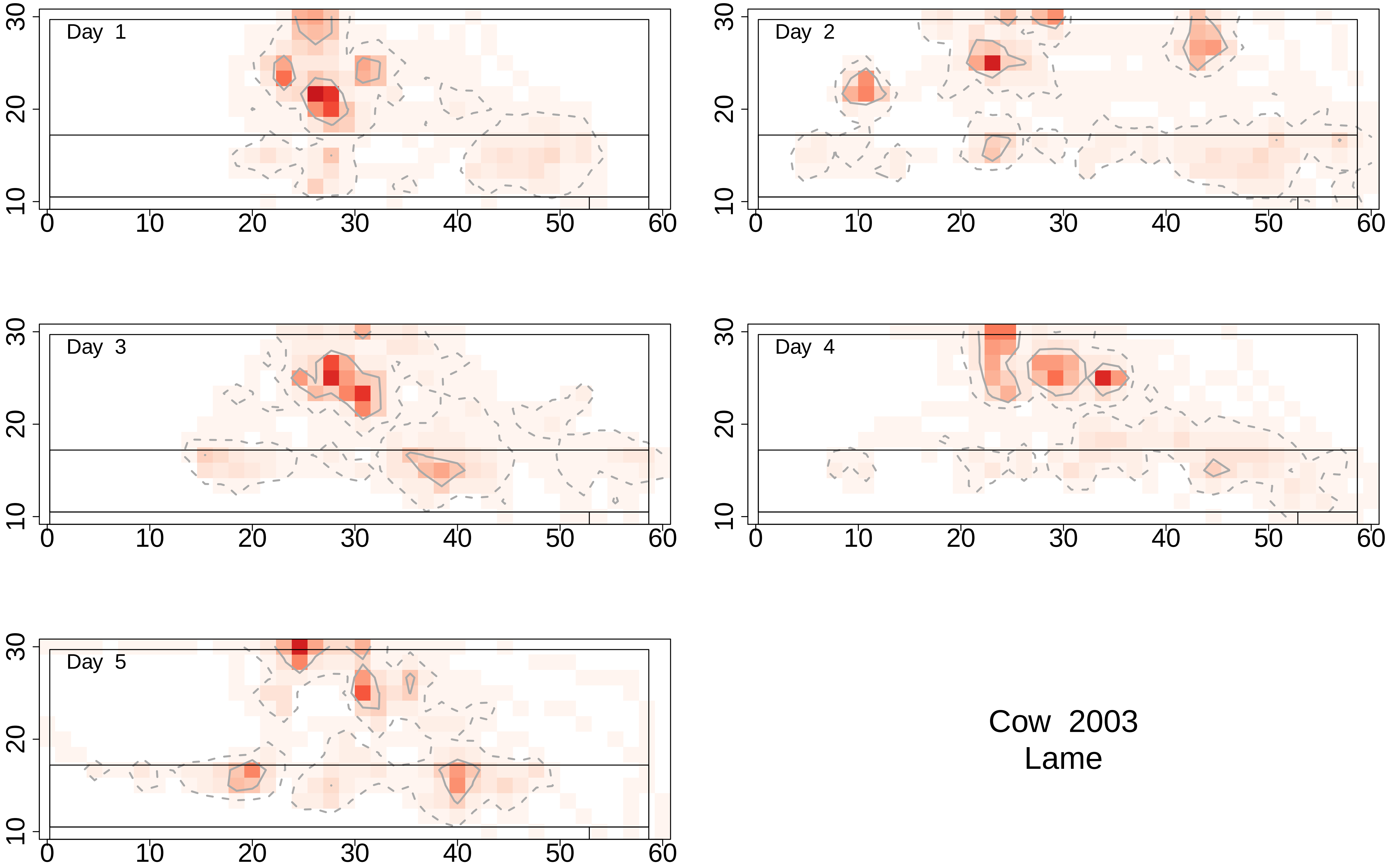

Day 1
Day 2
Day 3
Day 4
Day 5
0 10 20 30 40 50 60
10 20 30
Cow 2003
Lame

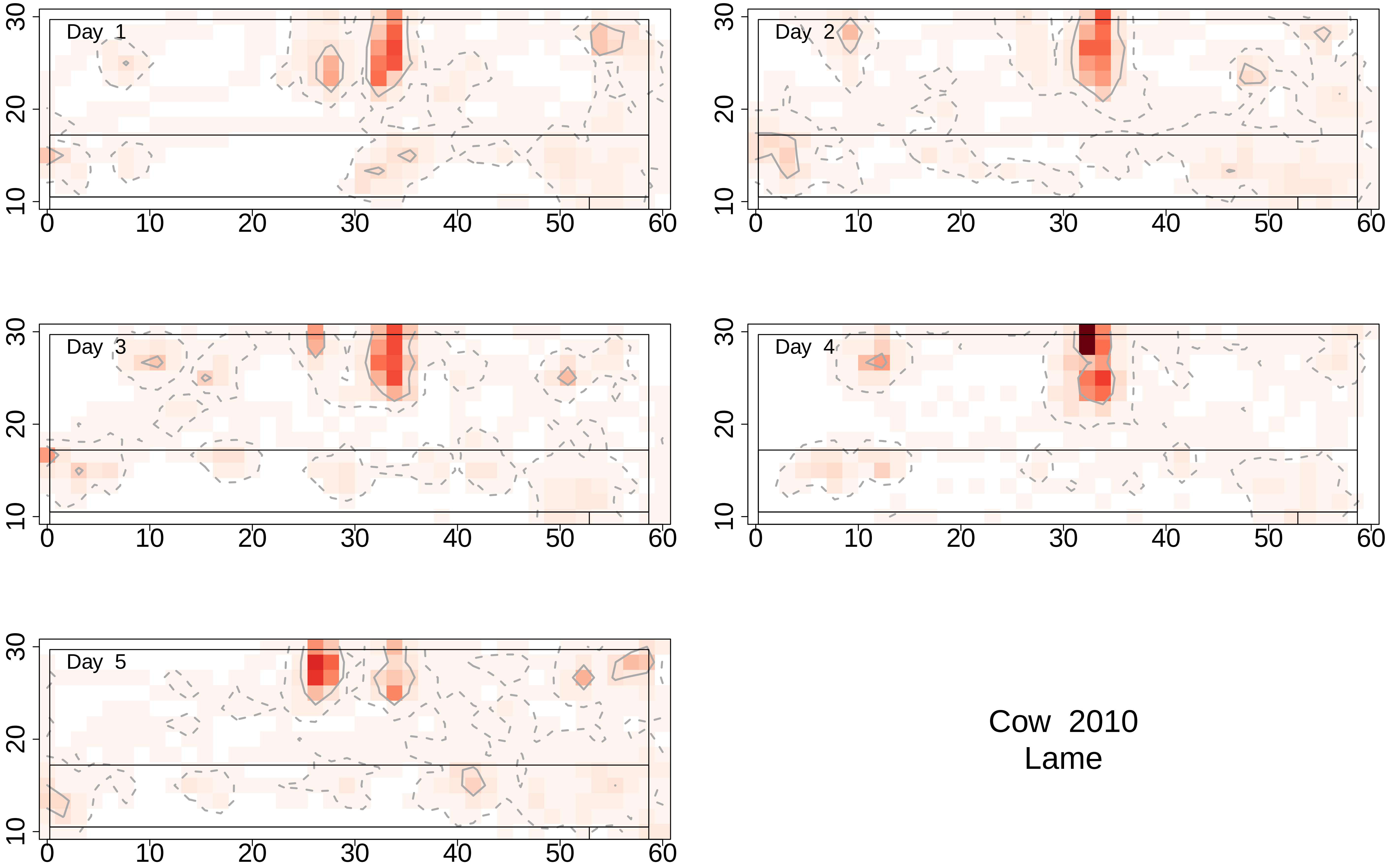
Day 1
Day 2
Day 3
Day 4
Day 5
Cow 2010
Lame

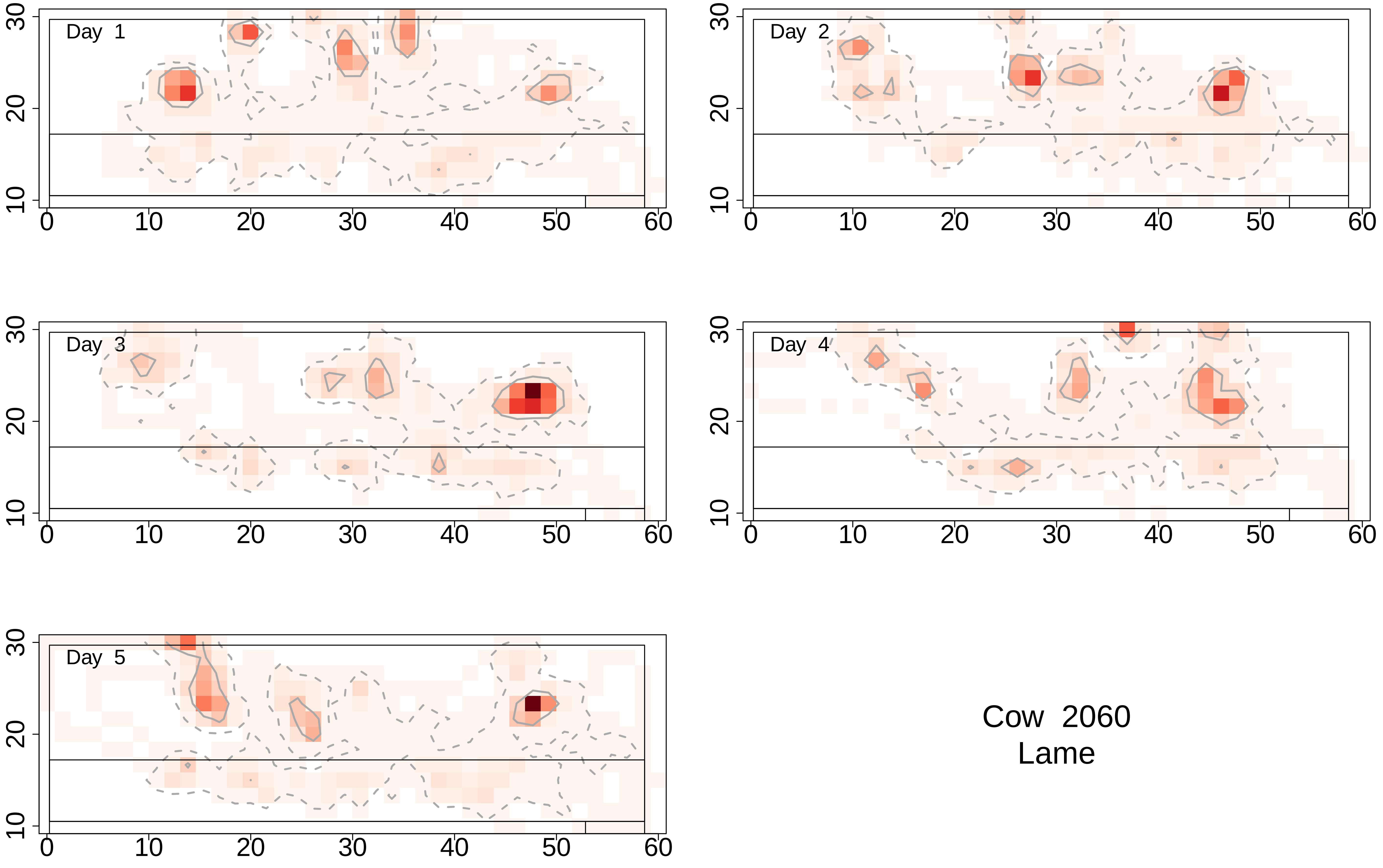
Day 1
Day 2
Day 3
Day 4
Day 5
0
10
20
30
40
50
60
Cow 2060
Lame

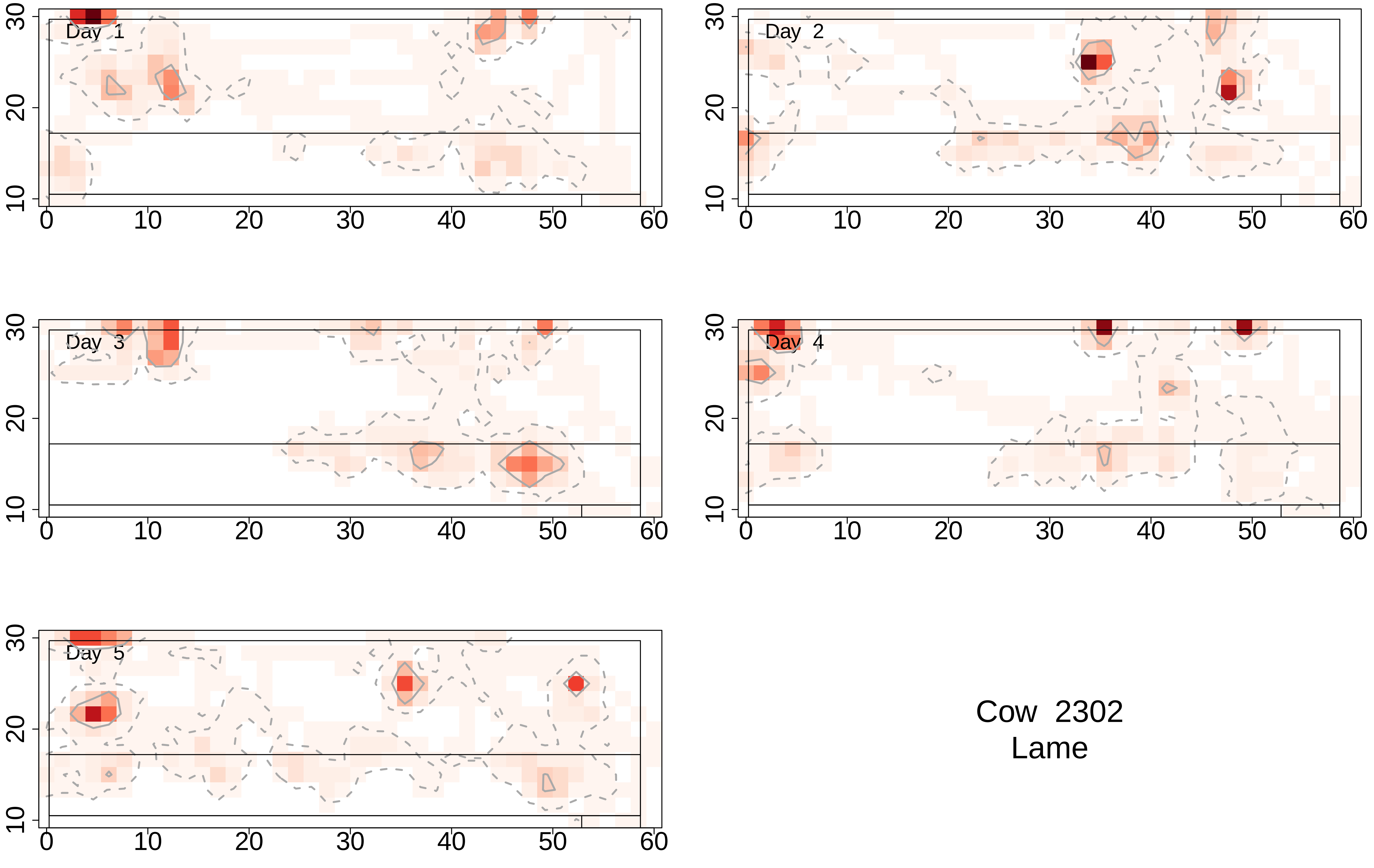
Day 1
Day 2
Day 3
Day 4
Day 5
0 10 20 30 40 50 60
10 20 30
Cow 2302
Lame

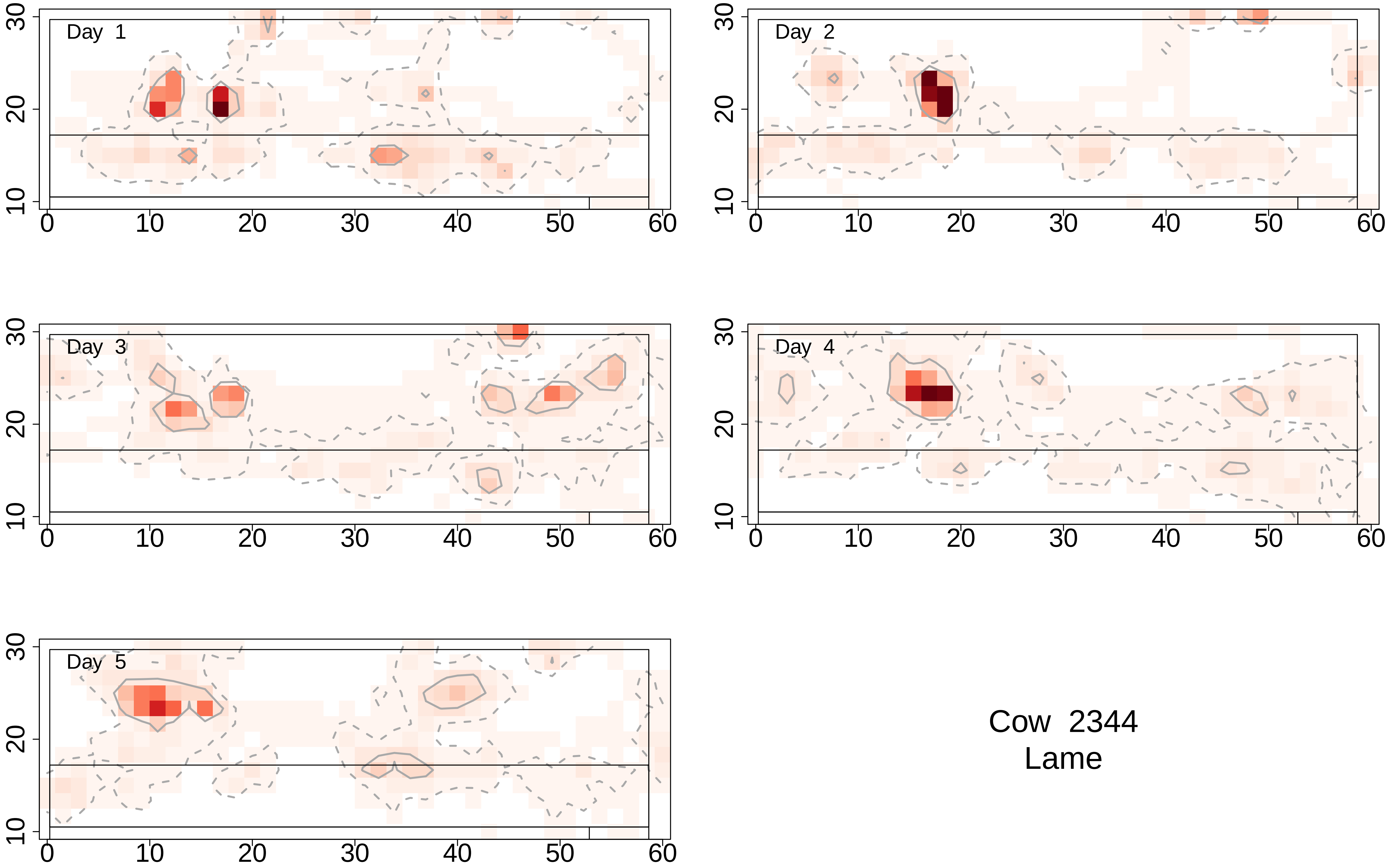

Day 1
Day 2
Day 3
Day 4
Day 5
0
10
20
30
40
50
60
10
20
30
Cow 2344
Lame

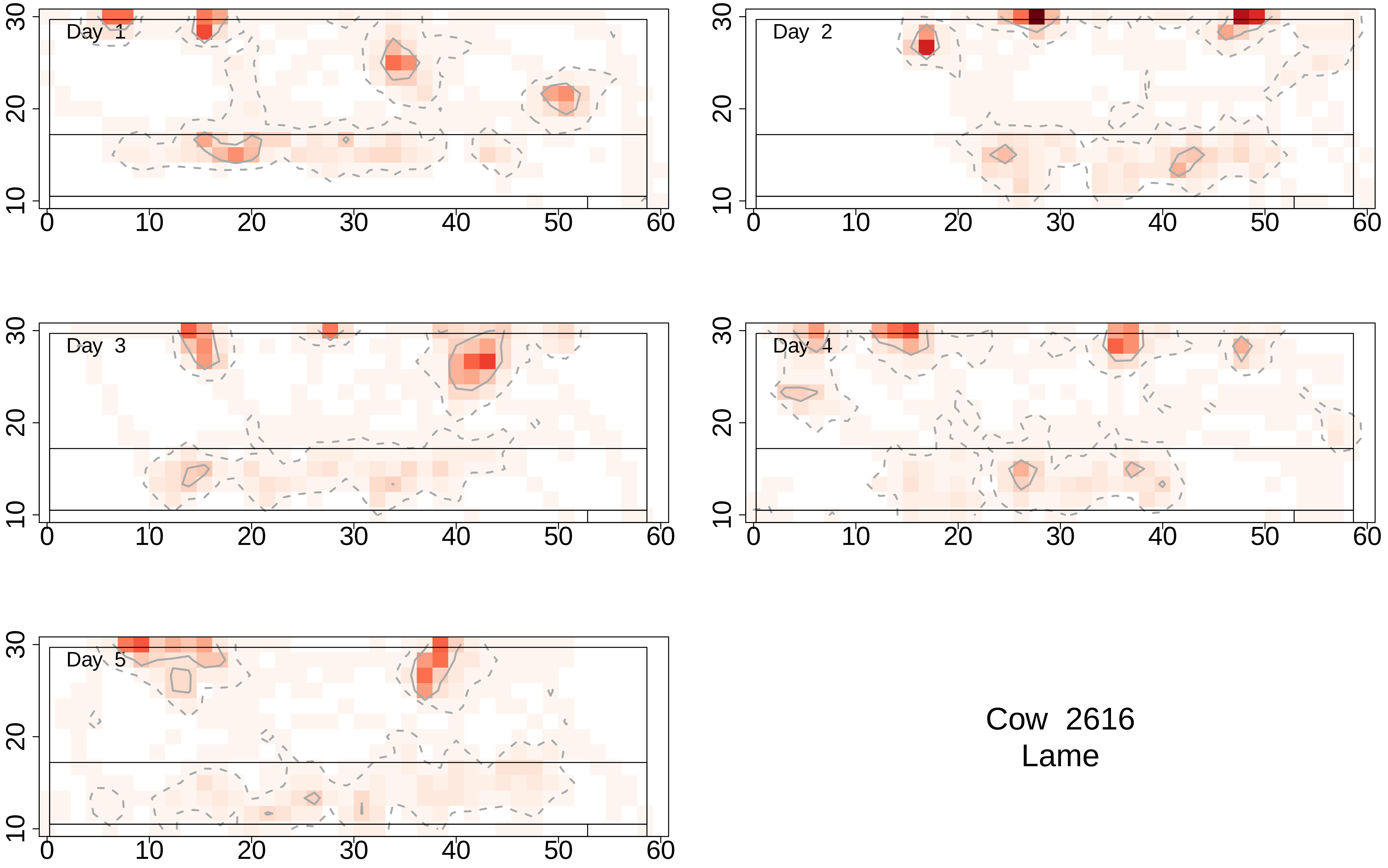

Day 1
Day 2
Day 3
Day 4
Day 5
Cow 2616
Lame

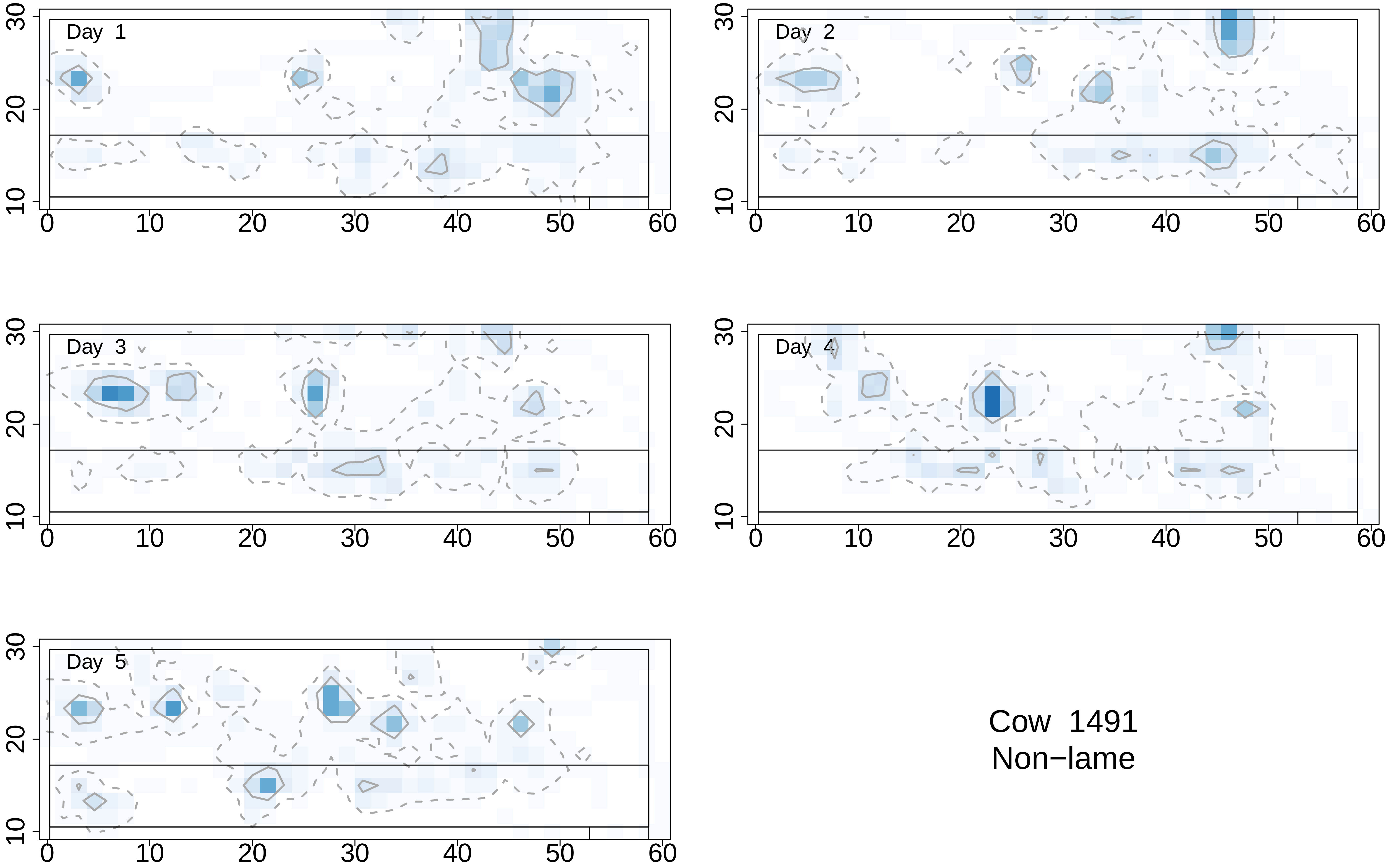
Day 1
Day 2
Day 3
Day 4
Day 5
Cow 1491
Non−lame

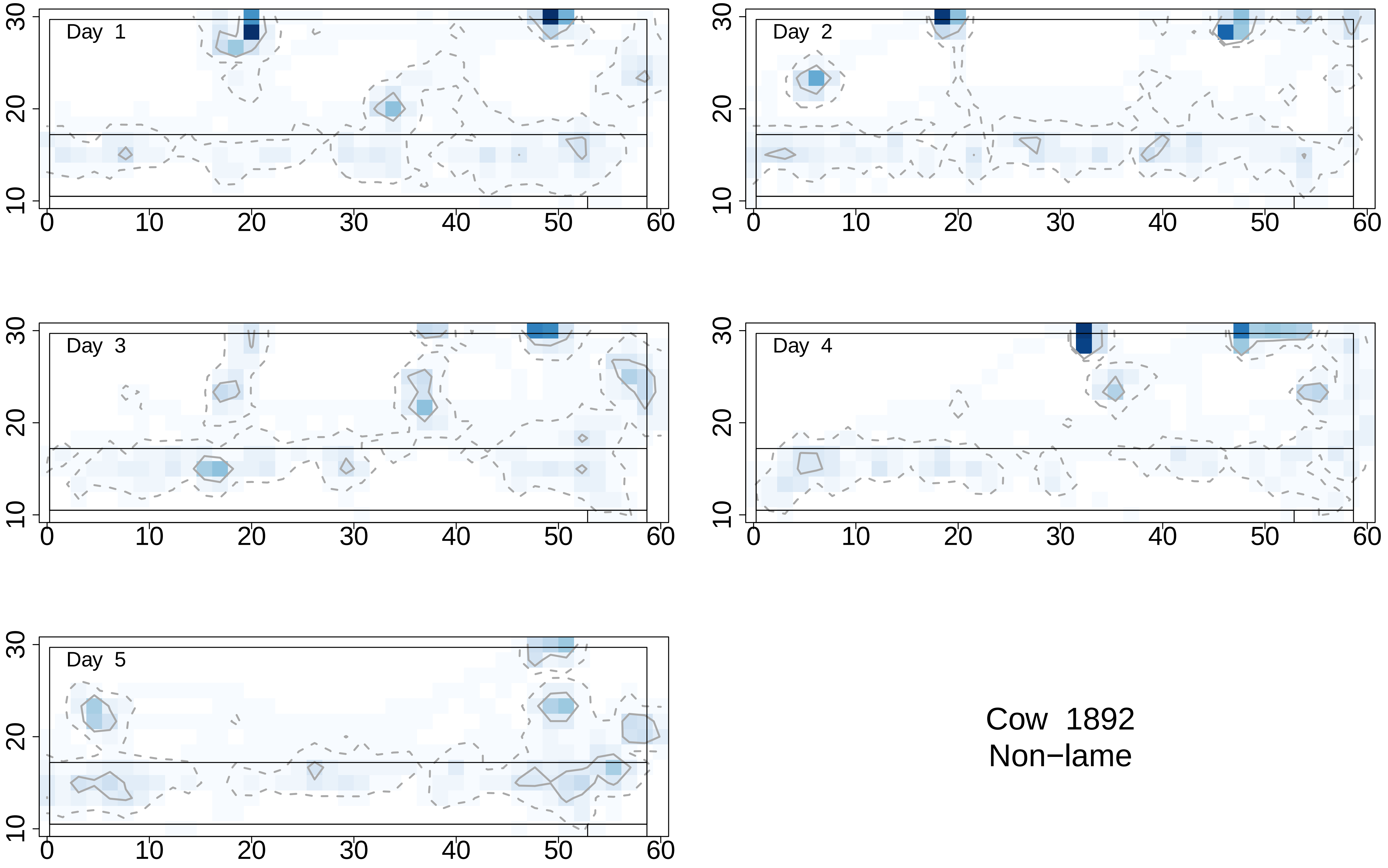

Day 1
Day 2
Day 3
Day 4
Day 5
Cow 1892
Non−lame

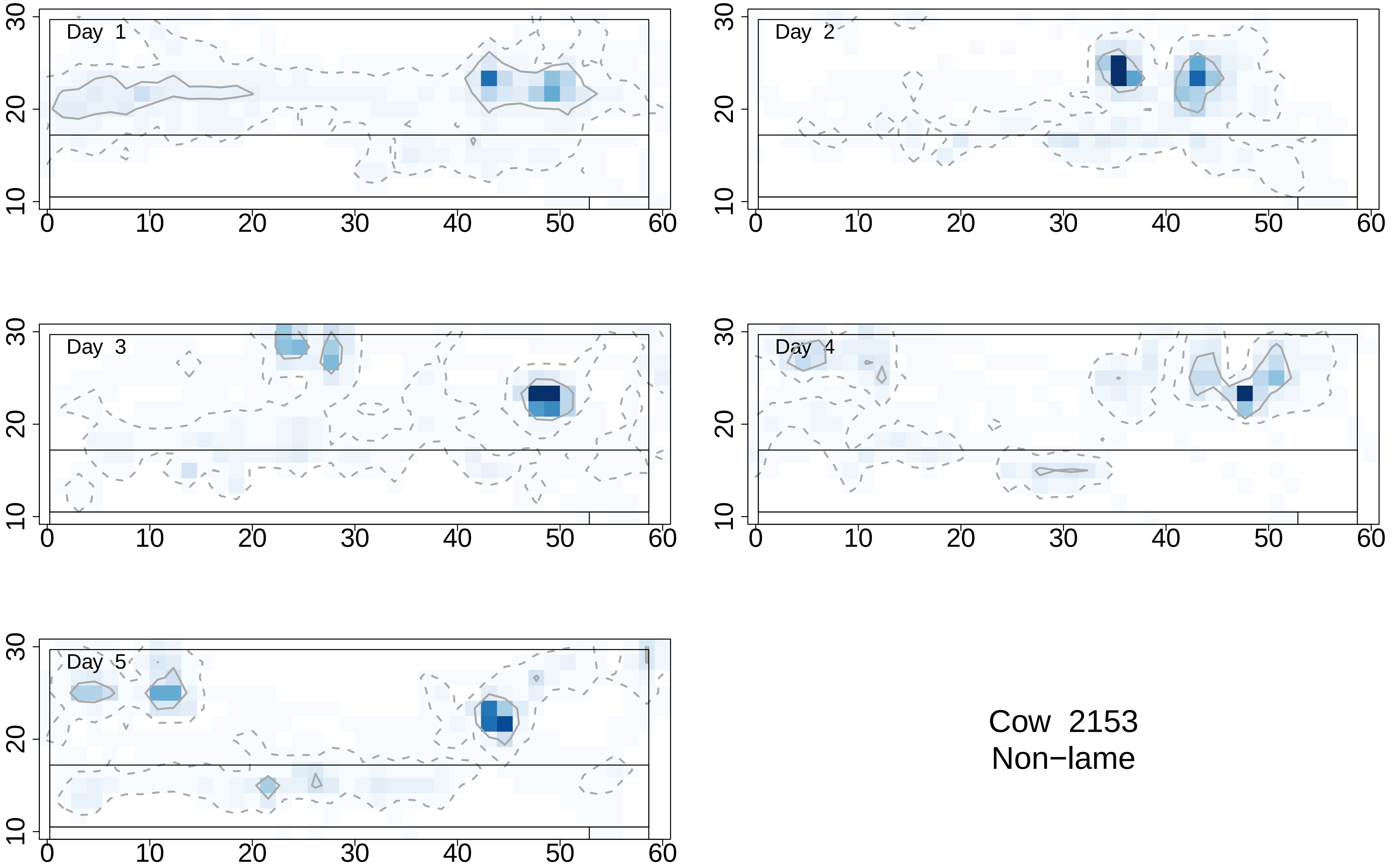
Day 1
Day 2
Day 3
Day 4
Day 5
Cow 2153
Non−lame

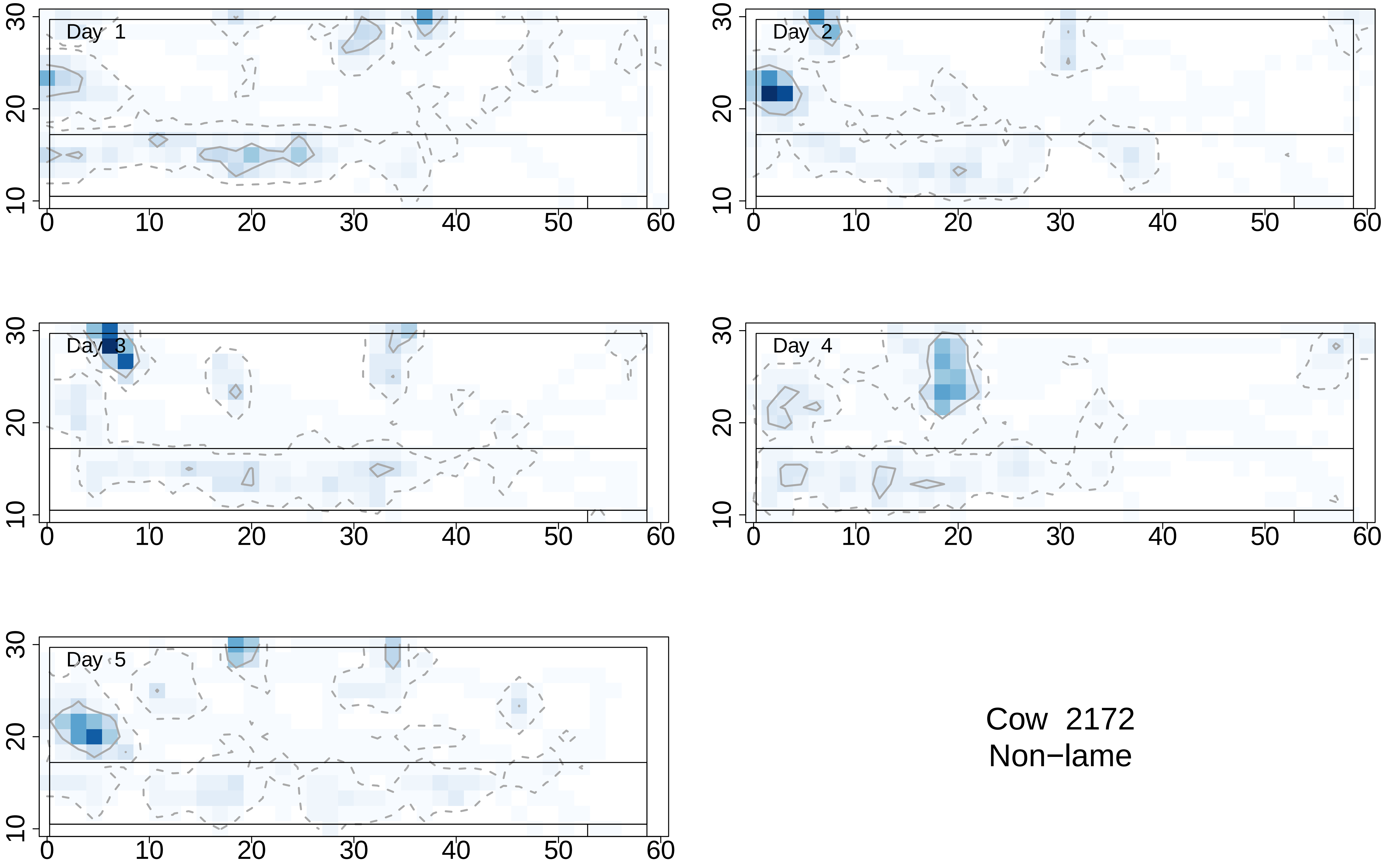
Day 1
Day 2
Day 3
Day 4
Day 5
Cow 2172
Non−lame

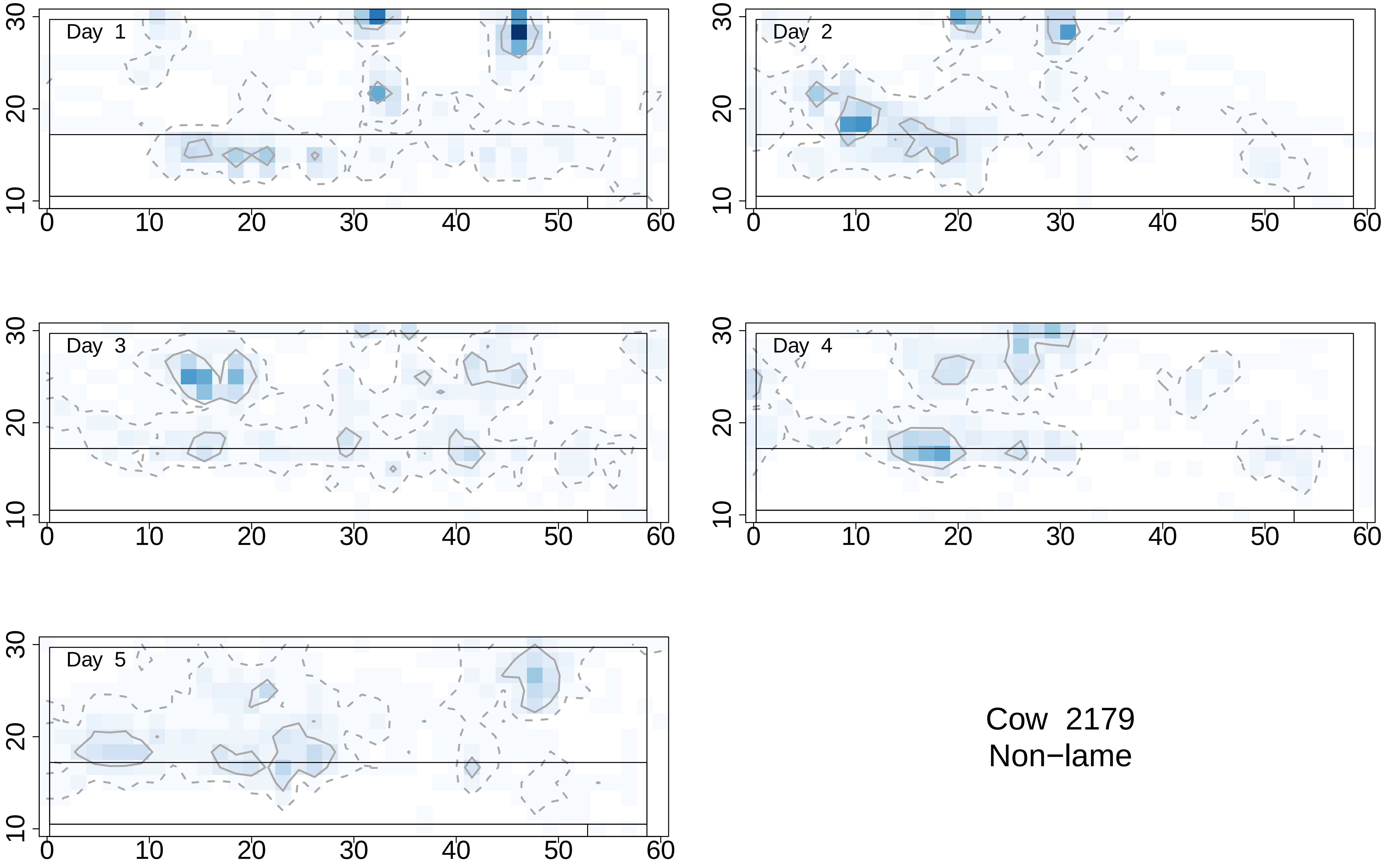

Day 1
Day 2
Day 3
Day 4
Day 5
0 10 20 30 40 50 60
10 20 30
Cow 2179
Non−lame

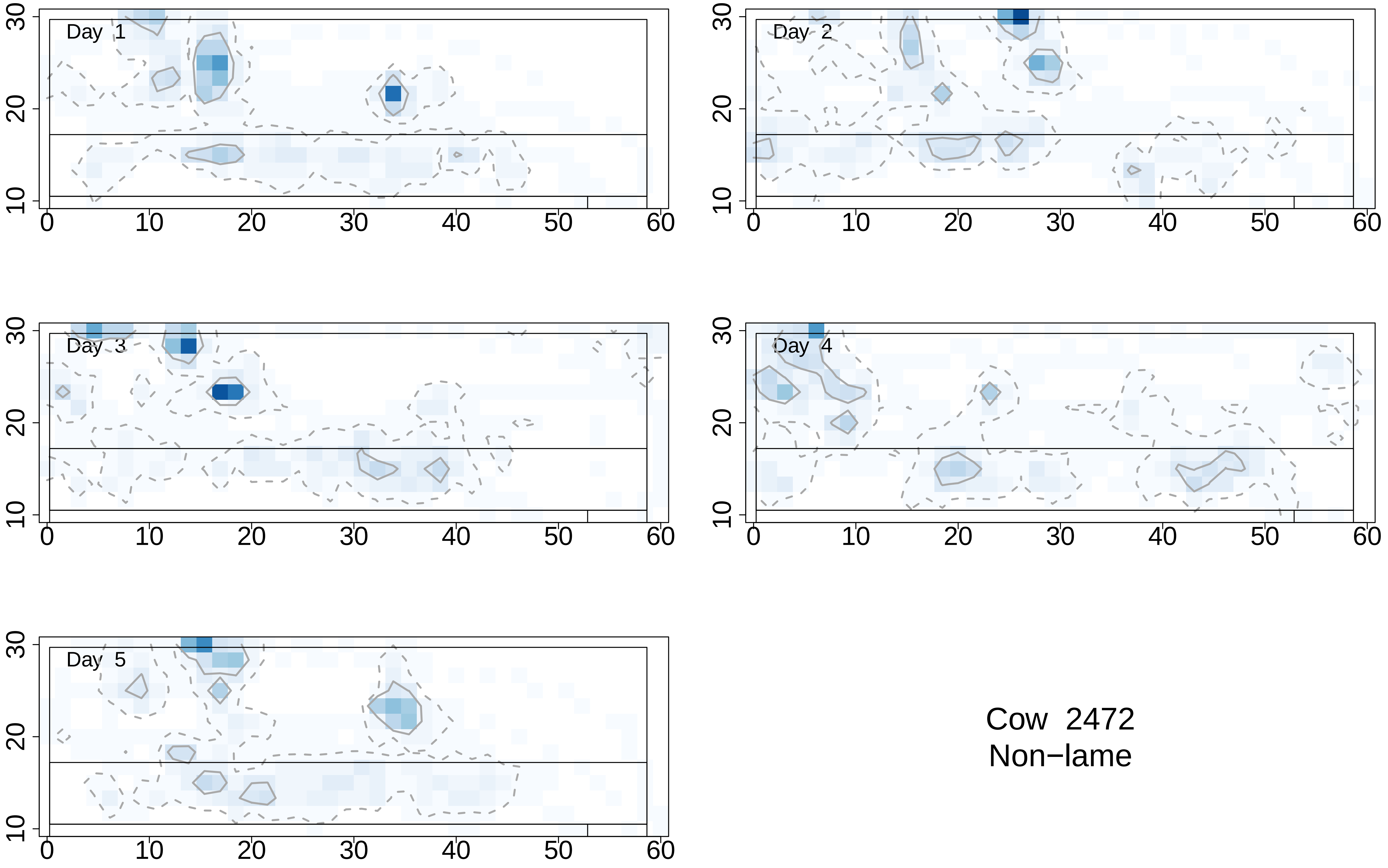

Day 1
Day 2
Day 3
Day 4
Day 5
0
10
20
30
40
50
60
10
20
30
Cow 2472
Non−lame

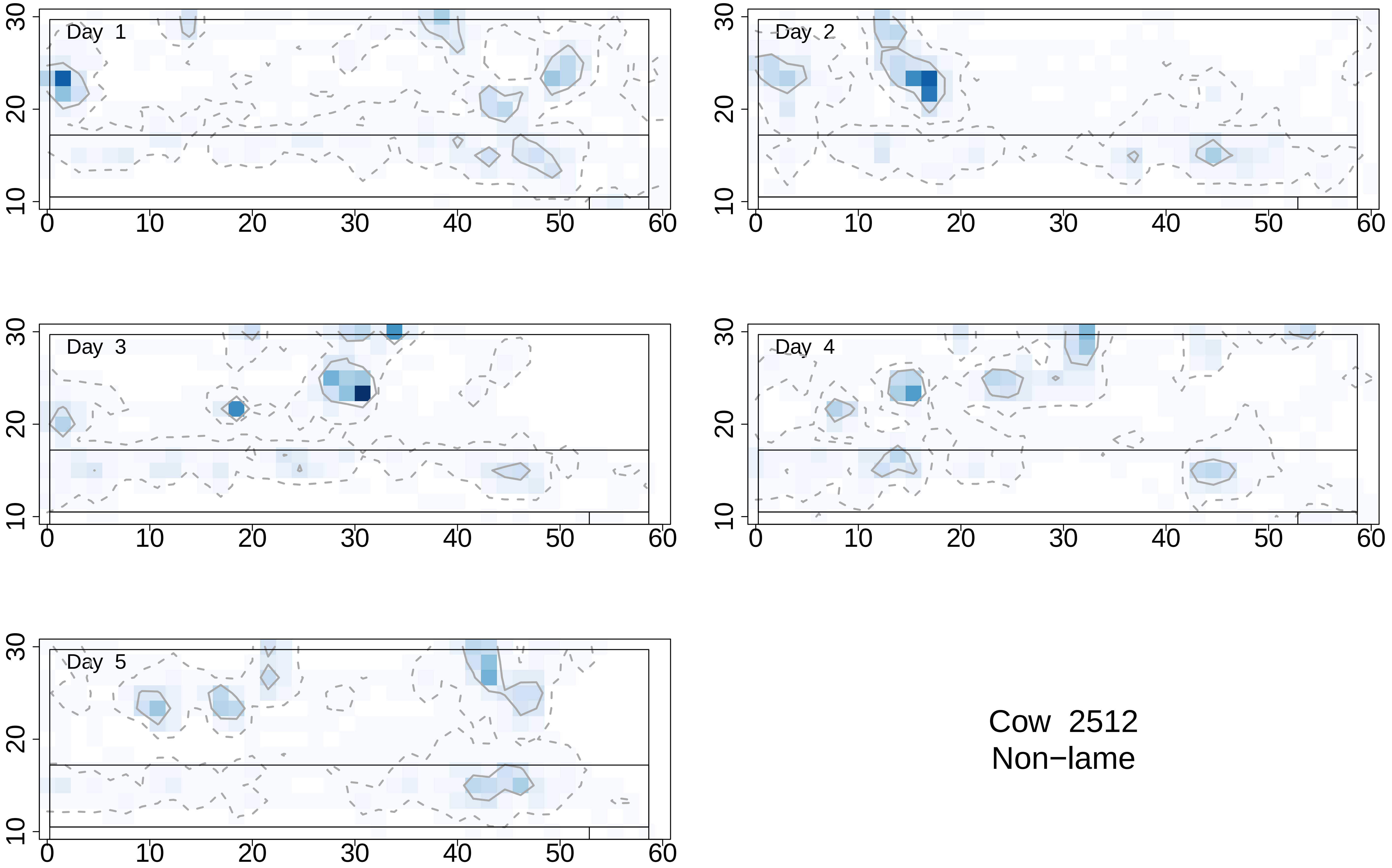

Day 1
Day 2
Day 3
Day 4
Day 5
10
20
30
0
10
20
30
40
50
60
Cow 2512
Non−lame

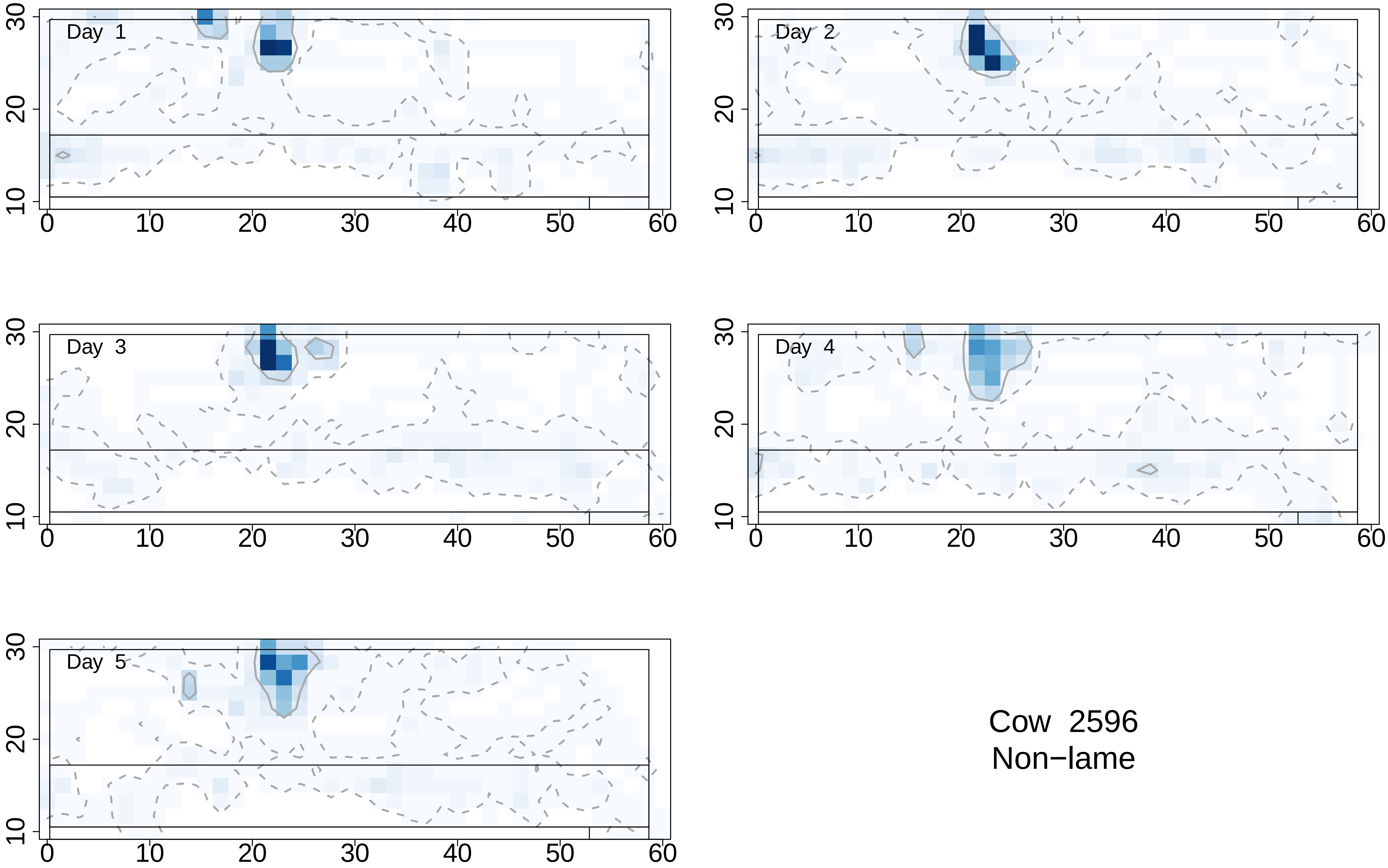

Cow 2596
Non−lame

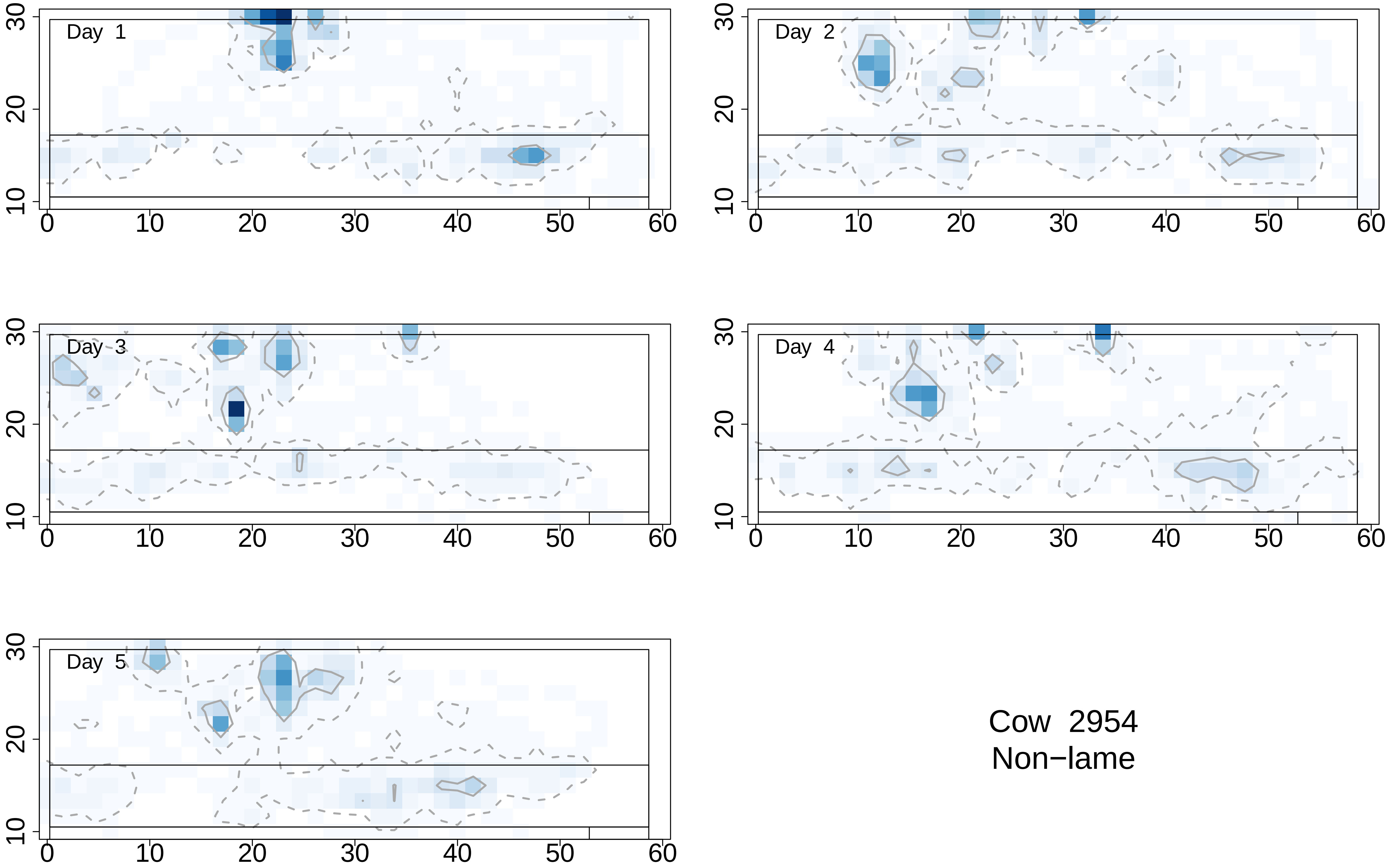
Day 1
Day 2
Day 3
Day 4
Day 5
Cow 2954
Non−lame

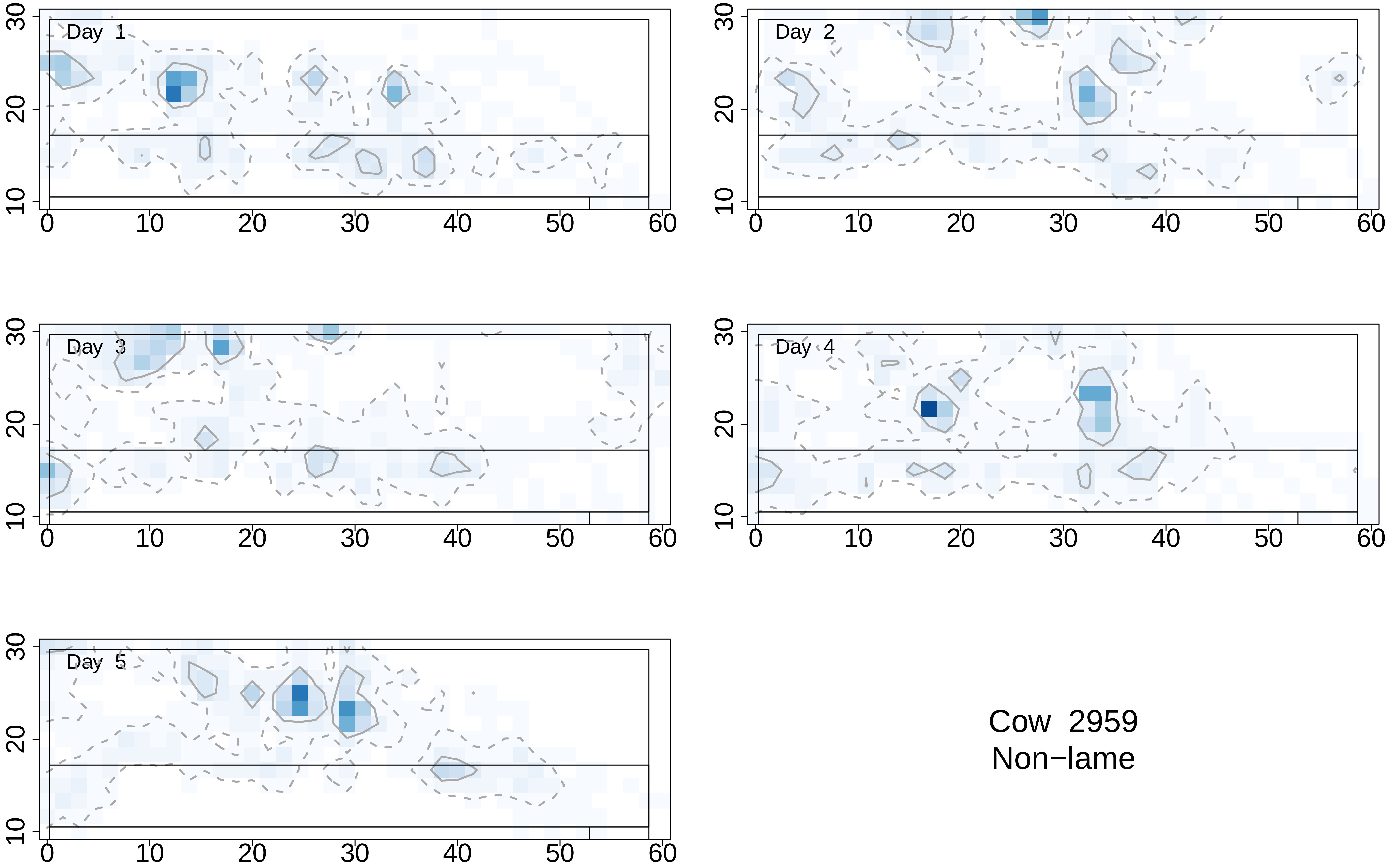
Day 1
Day 2
Day 3
Day 4
Day 5
10
20
30
0
10
20
30
40
50
60
Cow 2959
Non−lame
