# Supplementary figures and images for: Space-use patterns highlight behavioural differences linked to lameness, parity, and days in milk in barn-housed dairy cows
Source: PLoS One. 2018 Dec 19;13(12):e0208424. doi: 10.1371/journal.pone.0208424 (PMC6300209; doi:10.1371/journal.pone.0208424)

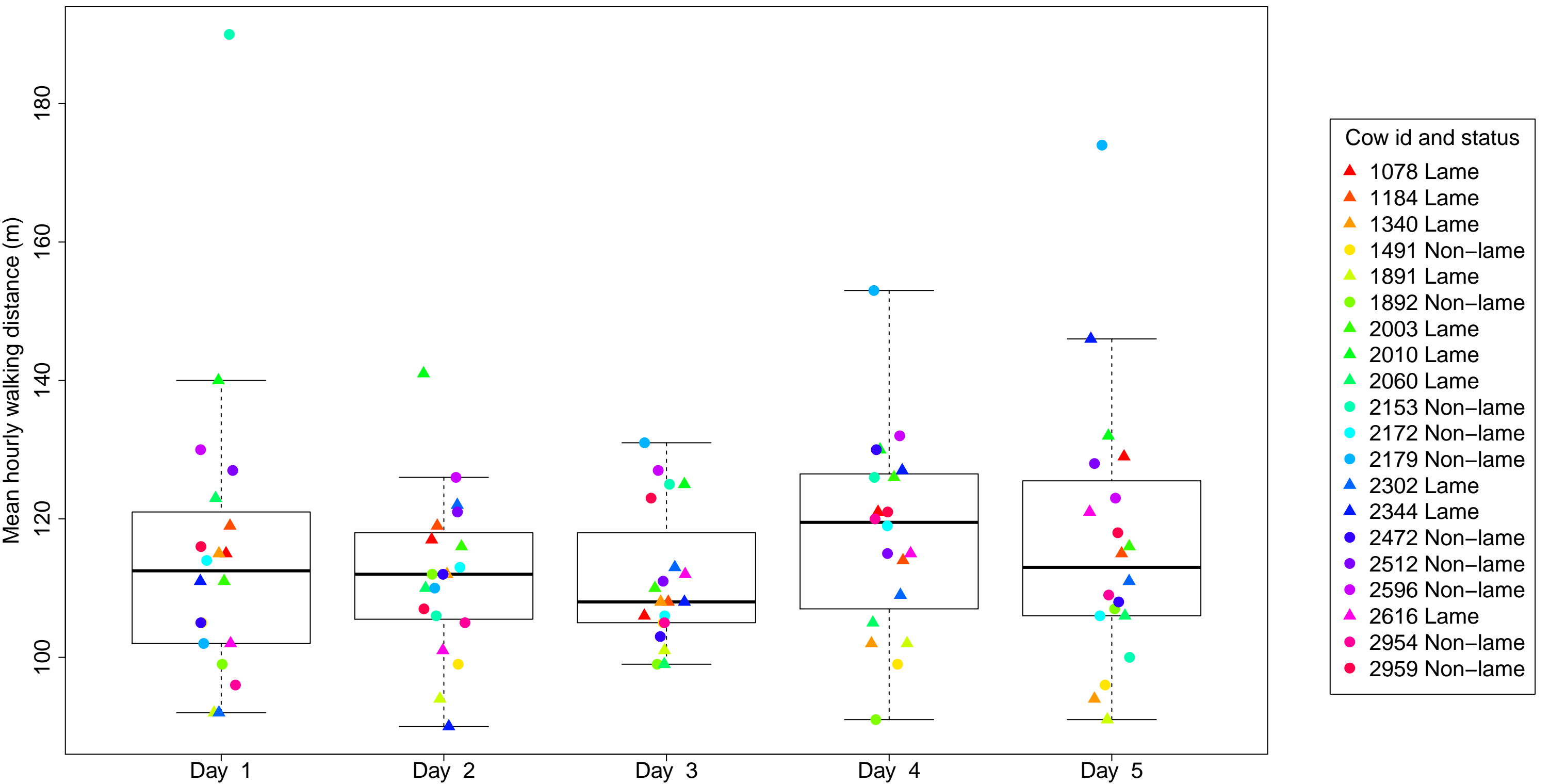

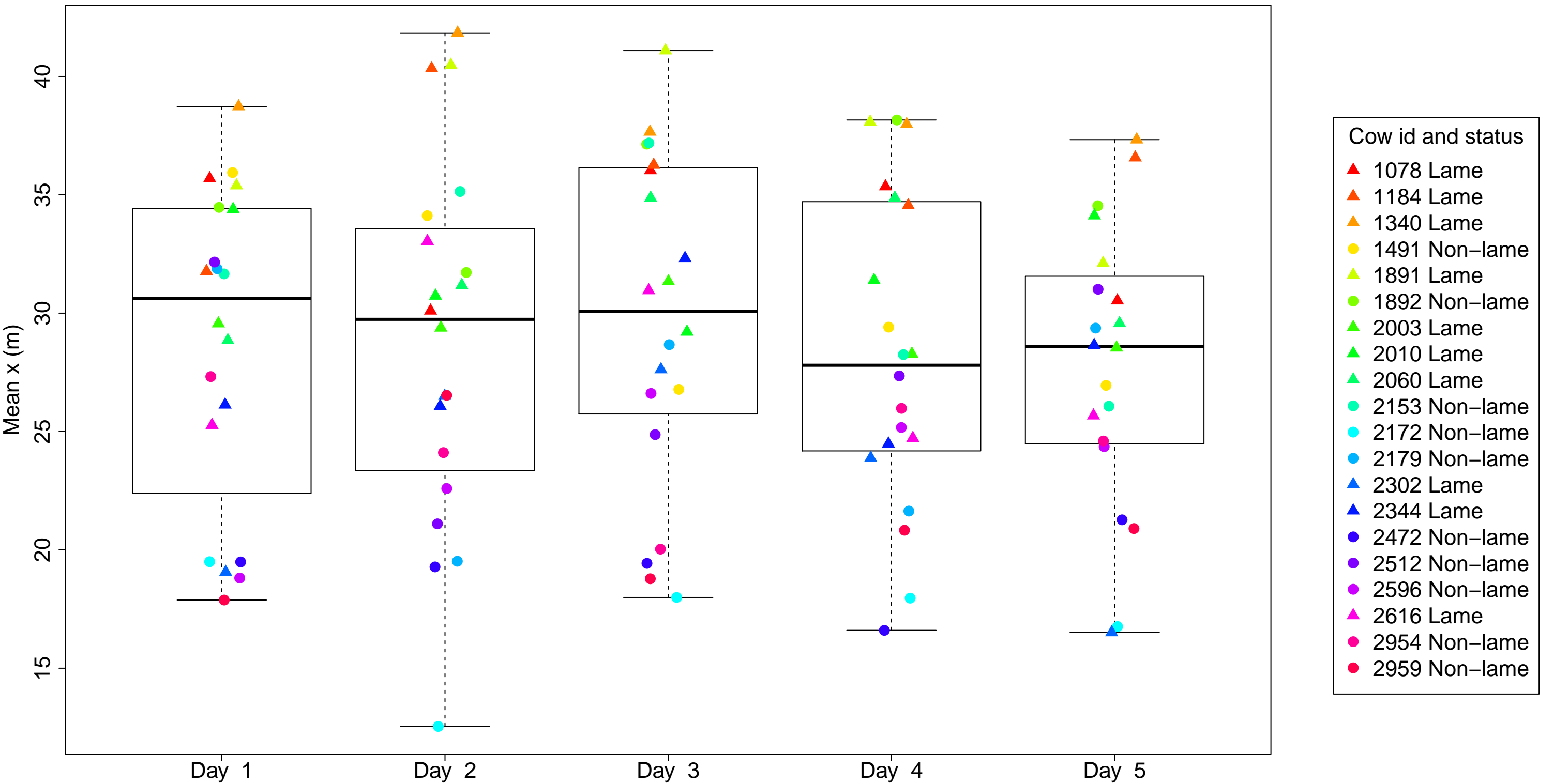

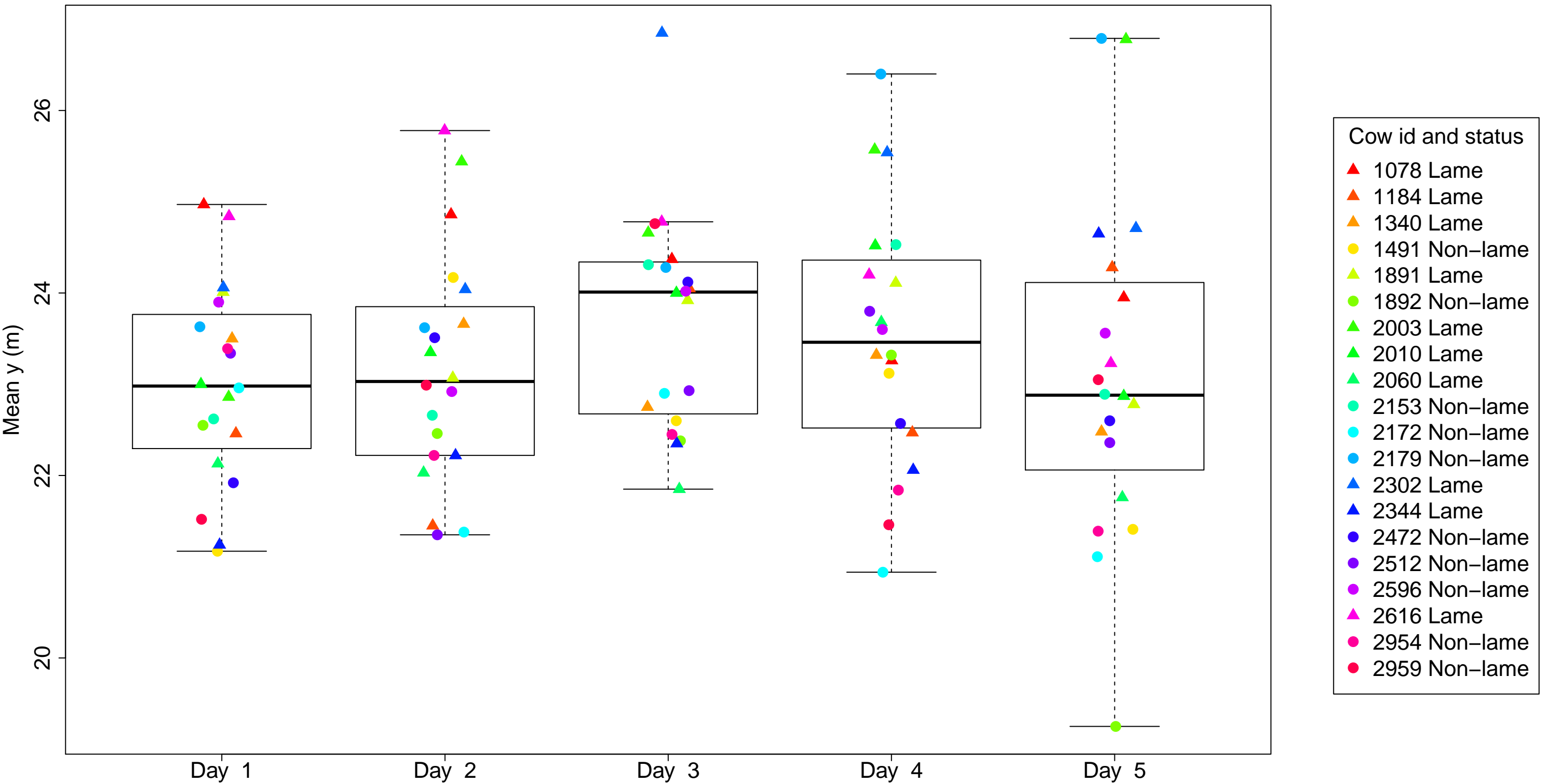

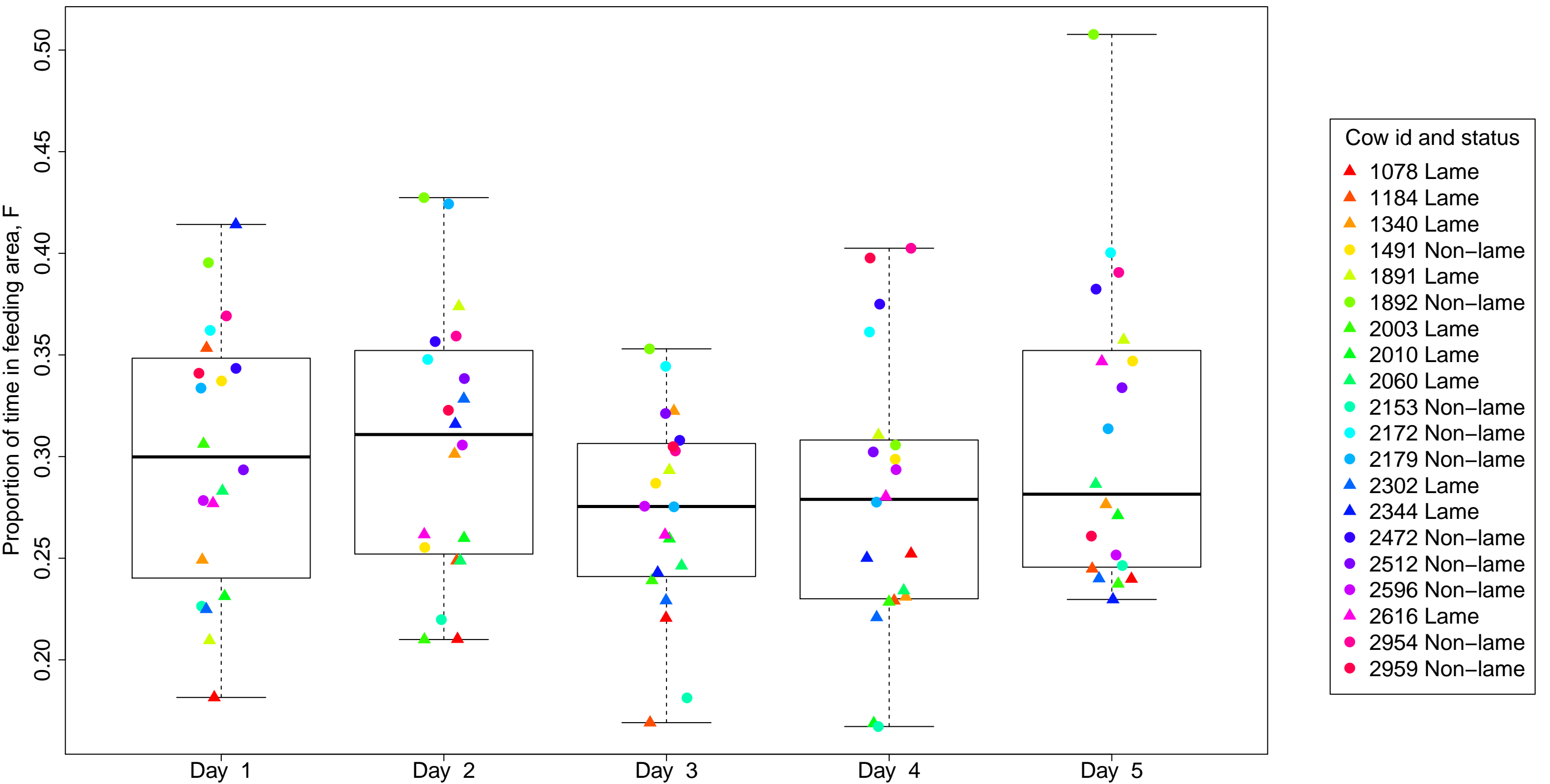

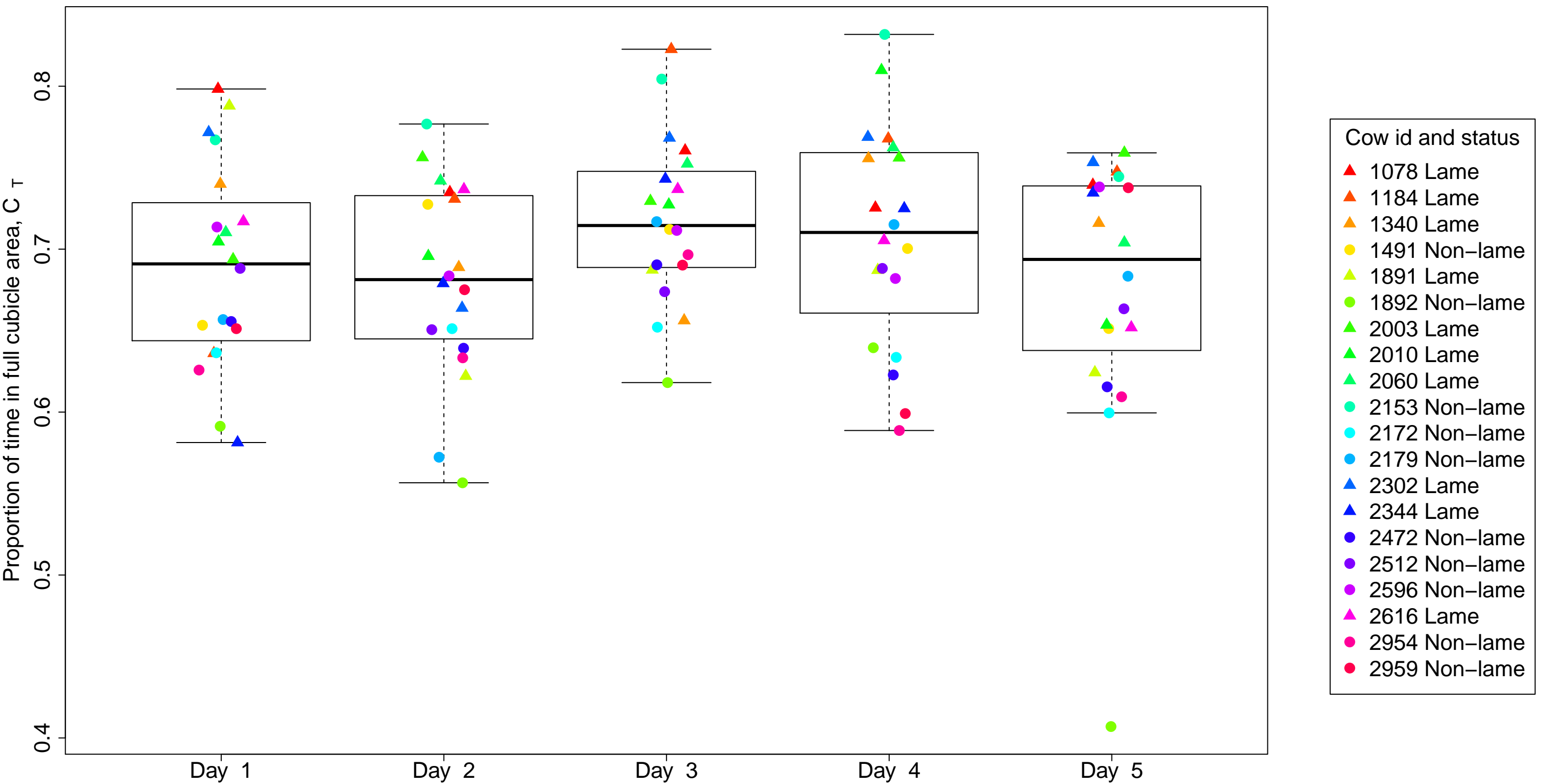

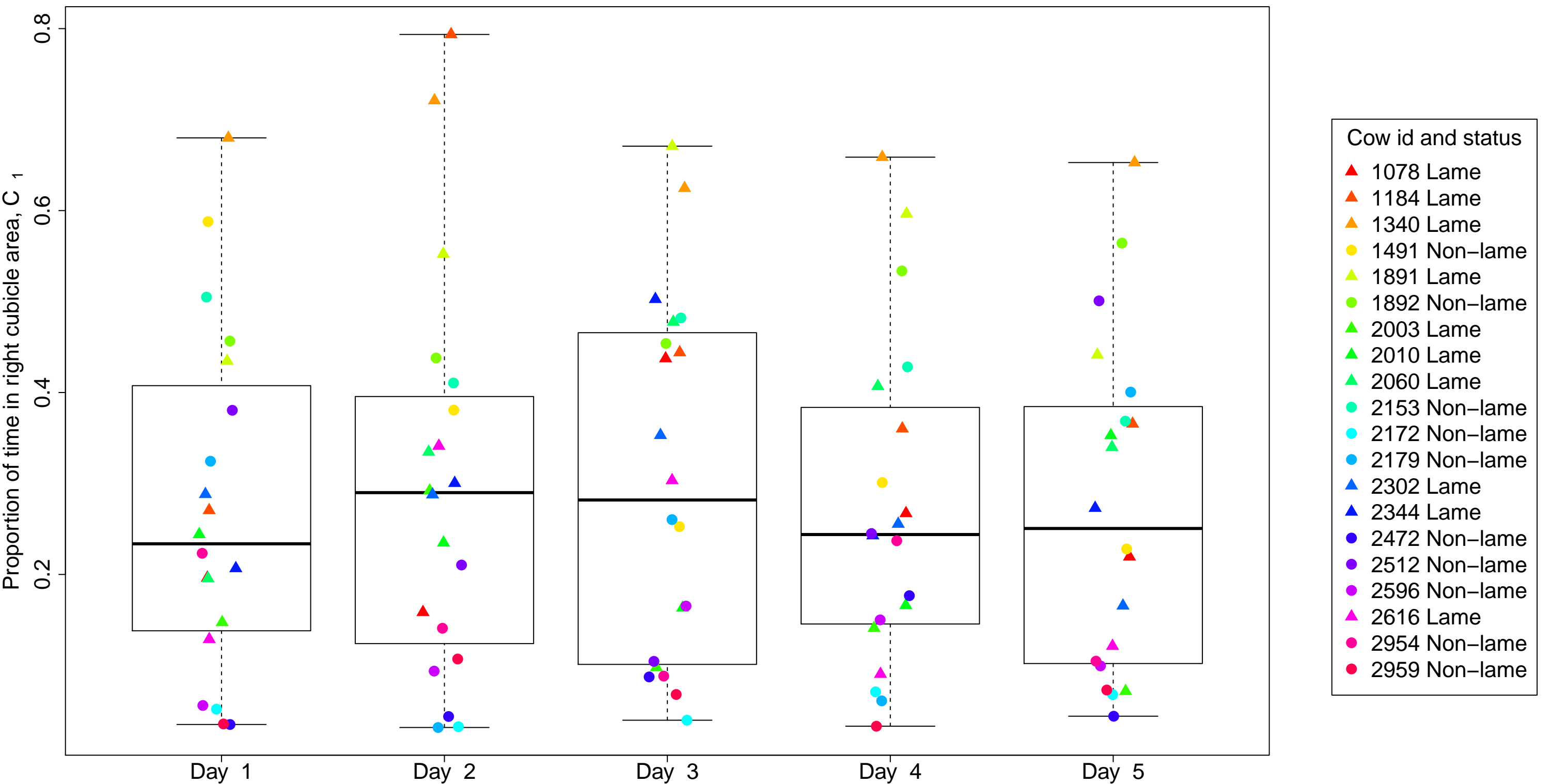

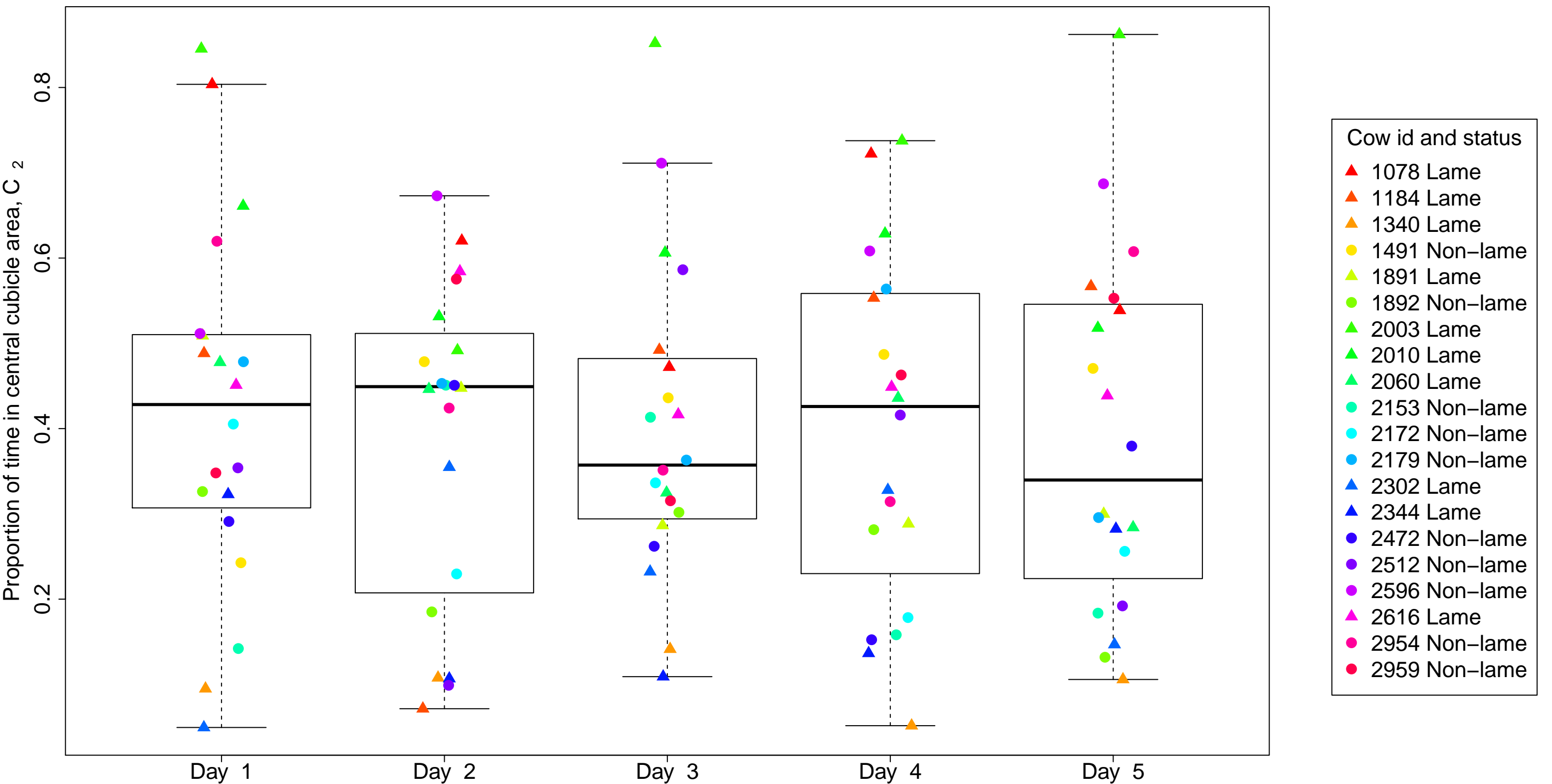

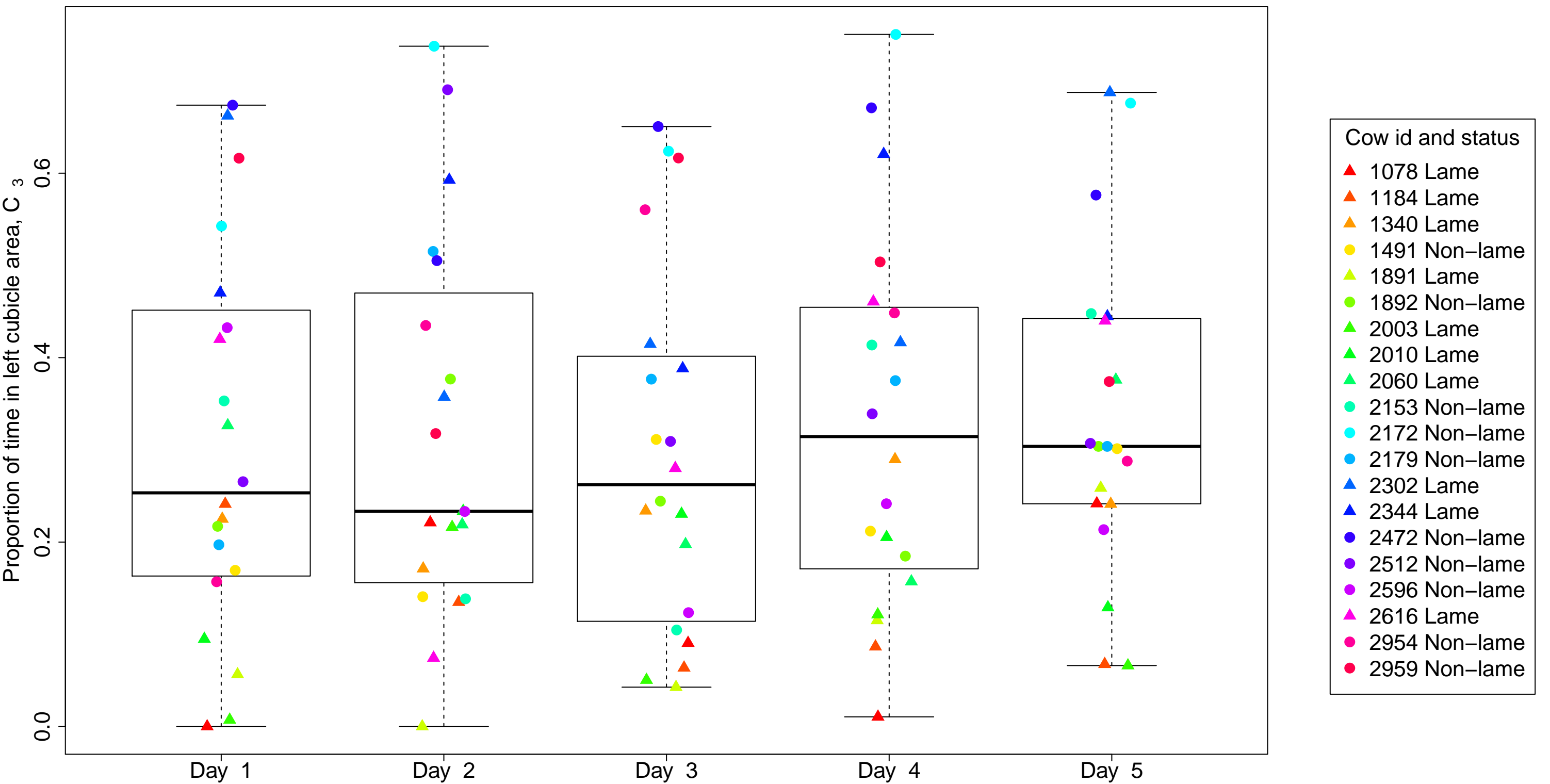

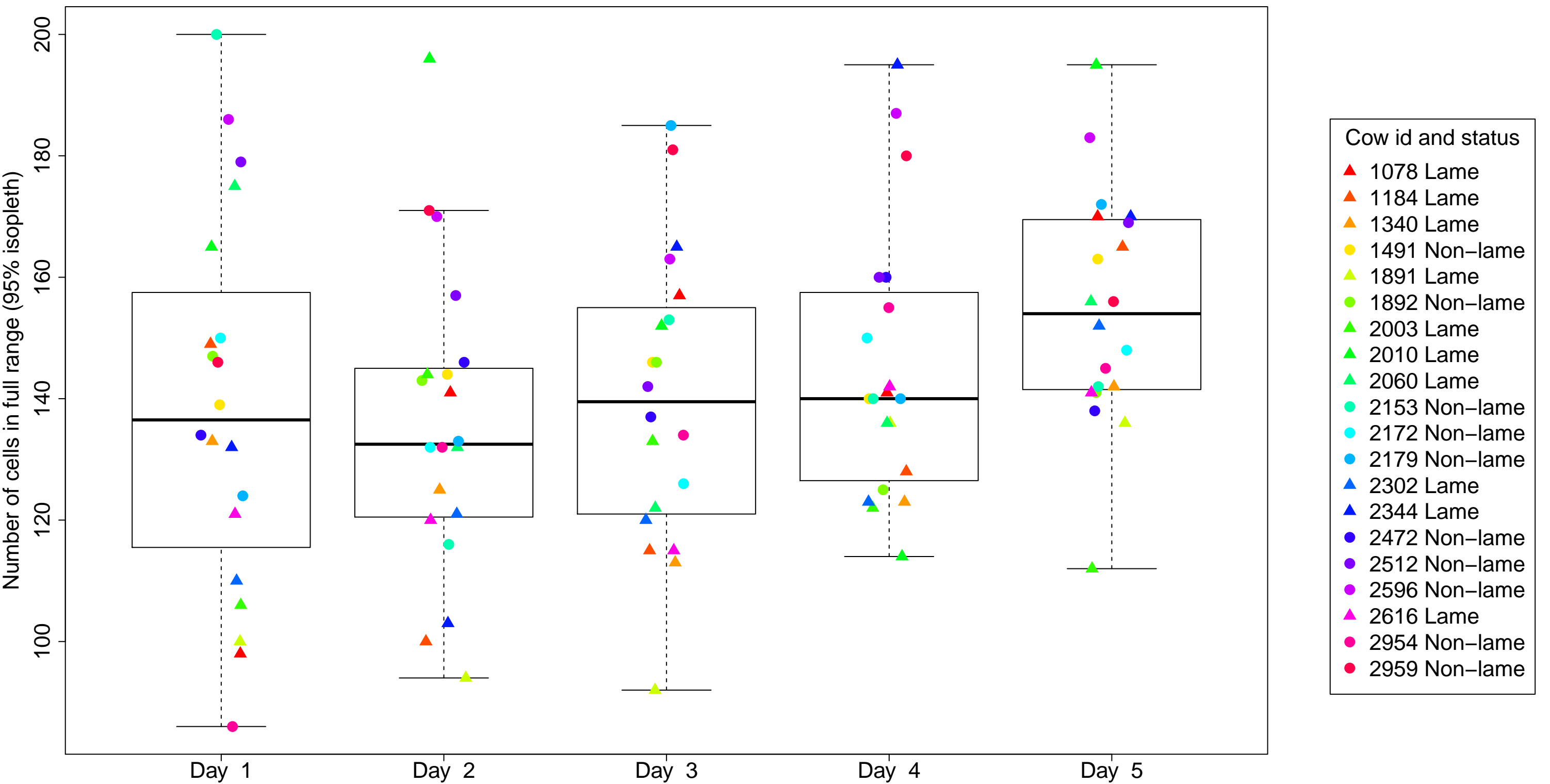

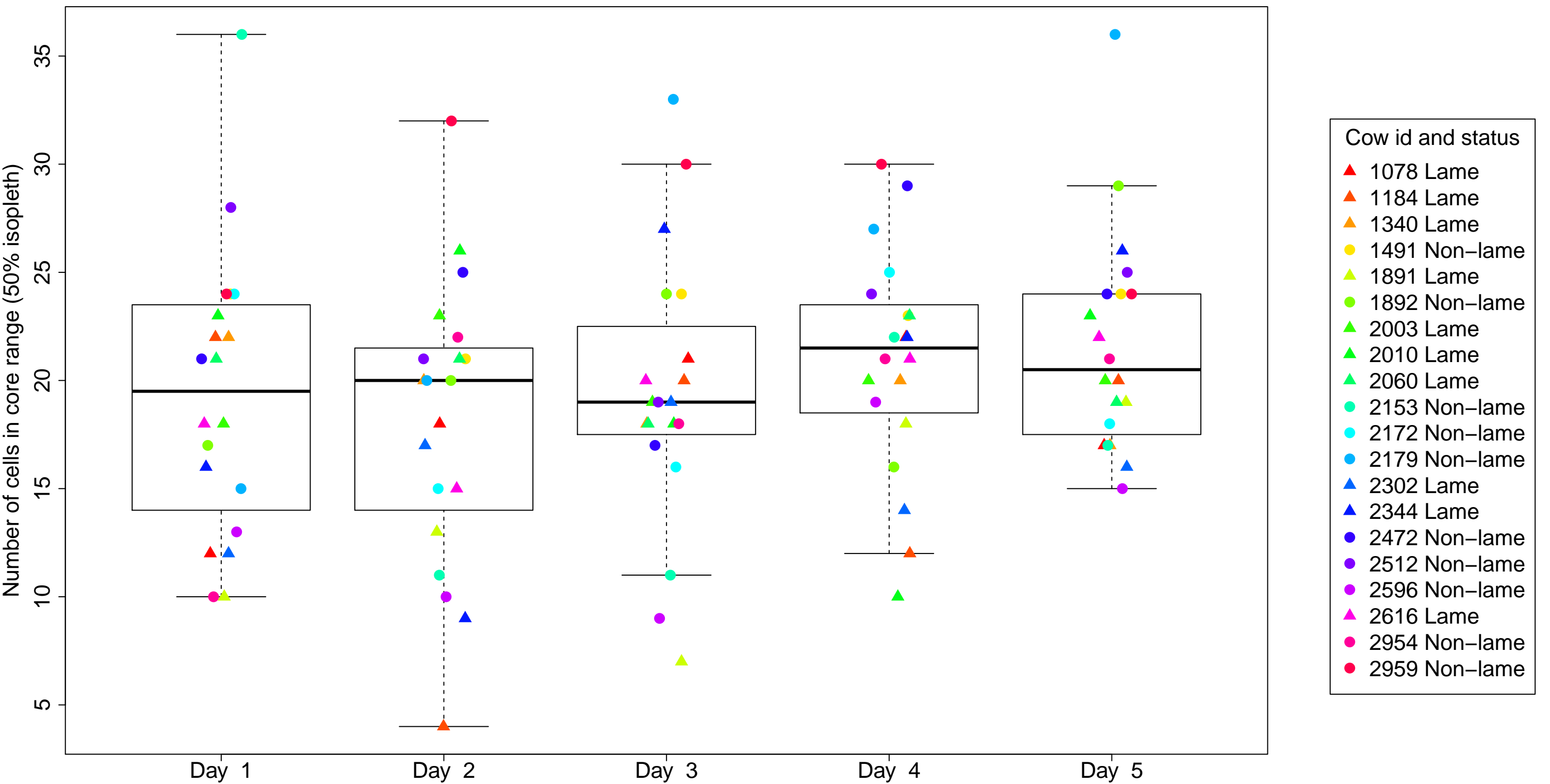

Supplement: S2 File — contains box-plots showing basic space-use measures by day of the trial. Lame cows are marked using triangles and non-lame cows are marked using circles. The colours used to indicate each cow are consistent across all plots. There are no clear trends by day in any of the basic space-use measures considered. (PDF) [file pone.0208424.s006.pdf]
